# Nanosilica Gel-Stabilized Phase-Change Materials Based on Epoxy Resin and Wood’s Metal

**DOI:** 10.3390/gels12010079

**Published:** 2026-01-16

**Authors:** Svetlana O. Ilyina, Irina Y. Gorbunova, Vyacheslav V. Shutov, Michael L. Kerber, Sergey O. Ilyin

**Affiliations:** 1 A.V. Topchiev Institute of Petrochemical Synthesis, Russian Academy of Sciences, 29 Leninsky Prospect, 119991 Moscow, Russia; 2 Department of Plastics Processing Technology, D. Mendeleev University of Chemical Technology of Russia, 9 Miusskaya Square, 125047 Moscow, Russia

**Keywords:** epoxy resin, silica nanoparticles, physical gelation, fusible alloys, Pickering emulsions, cross-linked gels, phase-change materials, rheology, calorimetry

## Abstract

The emulsification of a molten fusible metal alloy in a liquid epoxy matrix with its subsequent curing is a novel way to create a highly concentrated phase-change material. However, numerous challenges have arisen. The high interfacial tension between the molten metal and epoxy resin and the difference in their viscosities hinder the stretching and breaking of metal droplets during stirring. Further, the high density of metal droplets and lack of suitable surfactants lead to their rapid coalescence and sedimentation in the non-cross-linked resin. Finally, the high differences in the thermal expansion coefficients of the metal alloy and cross-linked epoxy polymer may cause cracking of the resulting phase-change material. This work overcomes the above problems by using nanosilica-induced physical gelation to thicken the epoxy medium containing Wood’s metal, stabilize their interfacial boundary, and immobilize the molten metal droplets through the creation of a gel-like network with a yield stress. In turn, the yield stress and the subsequent low-temperature curing with diethylenetriamine prevent delamination and cracking, while the transformation of the epoxy resin as a physical gel into a cross-linked polymer gel ensures form stability. The stabilization mechanism is shown to combine Pickering-like interfacial anchoring of hydrophilic silica at the metal/epoxy boundary with bulk gelation of the epoxy phase, enabling high metal loadings. As a result, epoxy shape-stable phase-change materials containing up to 80 wt% of Wood’s metal were produced. Wood’s metal forms fine dispersed droplets in epoxy medium with an average size of 2–5 µm, which can store thermal energy with an efficiency of up to 120.8 J/cm^3^. Wood’s metal plasticizes the epoxy matrix and decreases its glass transition temperature because of interactions with the epoxy resin and its hardener. However, the reinforcing effect of the metal particles compensates for this adverse effect, increasing Young’s modulus of the cured phase-change system up to 825 MPa. These form-stable, high-energy-density composites are promising for thermal energy storage in building envelopes, radiation-protective shielding, or industrial heat management systems where leakage-free operation and mechanical integrity are critical.

## 1. Introduction

In the modern world with worsening environmental conditions, the relevance of new technologies that allow for the efficient use of available energy resources is growing. Phase-change materials (PCMs) are one such technology that helps save energy, operating as an essential part of environmental improvements. In recent years, PCMs have found applications in various industries, such as construction [1,2,3], refrigeration and air conditioning systems [4,5], solar power systems [6,7], protective clothing [8], and medicine [9]. The demand and potential prospects for PCMs in these areas have initiated many advanced studies to improve their energy efficiency and expand their possible uses to other industrial fields and everyday life [10,11,12,13,14].

According to their nature, phase-change agents for creating PCMs can be organic [15], inorganic [16,17], and eutectic [18]. However, any new material creation, including a phase-change material, requires overcoming several problems. For example, organic PCMs have low thermal conductivity [19,20,21] and poor energy efficiency because of the incomplete crystallization of phase-change agents and their propensity for supercooling [22,23]. In turn, inorganic PCMs based on pure metals or salt hydrates have higher volumetric thermal energy storage capabilities, but they operate only at the high temperatures of their melting points [24]. In addition, they are prone to incomplete crystallization at a small size of the dispersed phase [25], which limits their wide application. At the same time, metal phase-change agents have the highest thermal conductivities and volumetric thermal energy storage capacities [26], attracting attention to address the problem of PCMs’ low thermal performance [27,28,29]. Moreover, a mix of different metals may produce eutectic alloys with lower melting points for various uses, including as phase-change agents. There are many fusible alloys of different compositions and melting points [30]. Still, the most available, cheapest, and well-known fusible alloy is Wood’s metal, which determined its selection as a model metallic phase-change agent.

A shape-keeping problem is specific to all phase-change agents during the transition from solid to liquid aggregate states at melting. The solution to the shape stability problem can be either encapsulating the phase-change agent in polymer shells [31,32] or placing it in a continuous matrix [33,34]. Because of the technological complexity of the encapsulation process, a direct dispersing of a phase-change agent in a matrix volume is a more straightforward method. In that case, the thermosetting epoxy resin is the most reasonable matrix material [35,36,37]. Thanks to its low viscosity, it is possible to realize uniform dispersing of phase-change agents in the form of particles or droplets in the whole volume of liquid epoxy medium, having good adhesion to substances of different natures after cross-linking. At the same time, the dispersion of phase-change agents in an emulsion form potentially allows for a higher packing density and lower energy consumption, thanks to the lower viscosities of emulsions than suspensions [38,39] (i.e., provides a chance to produce a more concentrated phase-change material with higher thermal performance).

However, the melt of any metal has a high surface tension [40], making its dispersing difficult in a low-viscosity epoxy medium. The formed droplets merge easily and, as a result, have a large size determined by the capillary number *Ca* achieved during mixing:

(1) $$Ca=\frac{\dot{\gamma }R\eta_{m}}{\xi },$$

where $\dot{\gamma }$ is the shear rate, *R* is the radius of the formed droplets, *η*_m_ is the viscosity of the continuous medium, and *ξ* is the interfacial tension, respectively [41].

For breaking up droplets because of their stretching under mixing, the capillary number must exceed a critical value that depends on the viscosity ratio of the dispersed phase and dispersion medium [42]. In addition, the density of metals is very high compared to the epoxy medium, which significantly accelerates the sedimentation of the resulting metal droplets. Therefore, when dispersing the phase-change agent, it is necessary to make the molten metal droplets resistant to coalescence and sedimentation. The way to kinetically slow down particle sedimentation is to reduce particle size, which requires (1) raising the mixing speed to increase the capillary number and consequently decrease droplet radius and (2) equal viscosities of the epoxy medium and liquid metal to minimize the critical capillary number necessary for droplet breakup. Furthermore, kinetic stability can be achieved by imparting a yield stress to the continuous medium through the addition of solid particles that form a physical gel, thereby immobilizing the emulsion droplets [43].

In the case of an epoxy medium, silica may act as an effective thickener that induces gel-like behavior, but preferably as nanoparticles to reduce their concentration and maintain a high concentration of a phase-change agent. Ordinary silica nanoparticles have a hydrophilic surface but can be chemically hydrophobized to improve affinity with organic matrices [44]. At the same time, it is unknown which type of silica particles will be best to act as a thickener of an uncured epoxy medium, especially at high temperatures of liquid metal emulsification, as heating changes the energies of both van der Waals interactions and hydrogen bonds between the components of the epoxy/silica mixture. Moreover, silica nanoparticles may act as Pickering stabilizers, forming mechanically robust barriers at liquid–liquid interfaces and thereby suppressing coalescence [45,46,47]. However, successful Pickering emulsification of molten metals remains rare, largely due to the scarcity of solid particles that are simultaneously (1) thermally stable at high temperatures, (2) interfacially active toward both metal and epoxy phases, and (3) compatible with subsequent thermoset curing. Therefore, it remains an open question whether unmodified hydrophilic or hydrophobic silica can fulfill these requirements in the Wood’s metal/epoxy system. In sum, the selection of nanosilica as the model particulate system for this study was driven by a unique combination of practical requisites that are not simultaneously fulfilled by other common fillers. Crucially, it is one of the very few—if not the only—industrially available nanomaterials that is offered in both well-defined hydrophilic and hydrophobic forms, enabling a direct comparison of surface chemistry effects. This dual availability is essential for a foundational study where the optimal wettability for metal/epoxy interfaces was unknown. Furthermore, its nanoscale primary particle size (7–16 nm) permits the formation of a space-filling network at low concentrations (<15 wt% [48]), maximizing the volume fraction for the phase-change agent, while its high thermal stability (>200 °C) exceeds the processing window of low-melting-point alloys. Alternative candidates, such as organoclays (e.g., montmorillonite), often derive their hydrophobicity from surfactants that can leach into the epoxy matrix, potentially compromising interfacial integrity and cure kinetics. Thus, silica presented itself as the only chemically robust and systematically variable option to test the central hypothesis of this work.

Another problem with encapsulated or dispersion-filled PCMs is the difference in the thermal expansion coefficients of a phase-change agent and a polymer shell or matrix [49,50]. Any metal has a lower coefficient of thermal expansion than the epoxy matrix, which may lead to cracking of the produced material during melting and crystallization cycles. To avoid that, the curing of metal-containing epoxy materials should be at low temperatures. In this case, the subsequent heating of the resultant cross-linked PCM would lead to a more intense expansion of the epoxy matrix relative to the dispersed metal droplets, allowing for the mechanical integrity of the system to be maintained. Most available low-temperature epoxy hardeners are aliphatic amines, such as diethylenetriamine (DETA) [51,52], predetermining its selection for research.

Previous works have considered metals as thermally conductive particles [53,54,55,56] or open-cell foams to enhance the thermal conductivity of PCMs [57,58,59]. Metals and fusible alloys have also acted as phase-change agents, their components, and thermally conductive shells for PCM capsules [60,61,62,63,64,65,66]. However, the literature contains limited data on PCMs with a dispersed metal phase-change agent and a continuous polymer medium [29,67], which can result from the difficulty in efficiently dispersing a metal in a polymer medium to obtain highly filled systems.

This work aims to get a phase-change material based on epoxy resin, DETA, and Wood’s metal, stabilized from coalescence and settling with solid silica nanoparticles of a hydrophilic or hydrophobic nature. The initial stage of the present study examines the rheological behavior of epoxy resin containing silica nanoparticles of a hydrophilic or hydrophobic nature to determine their gel-forming ability at different temperatures. The study then focuses on dispersions of Wood’s metal in an epoxy matrix at various temperatures in the presence of silica nanoparticles introduced to impart yield stress to the system and thereby ensure stability against coalescence, aggregation, and sedimentation. The study concludes by evaluating the influence of Wood’s metal on the morphology, mechanical properties, and thermophysical characteristics of the final phase-change materials whose shape stability stems from the transformation of the metal-containing epoxy physical gels into cross-linked polymer networks.

## 2. Results and Discussion

### 2.1. Size Distribution of Silica Nanoparticles in Epoxy Resin

**Figure 1:**
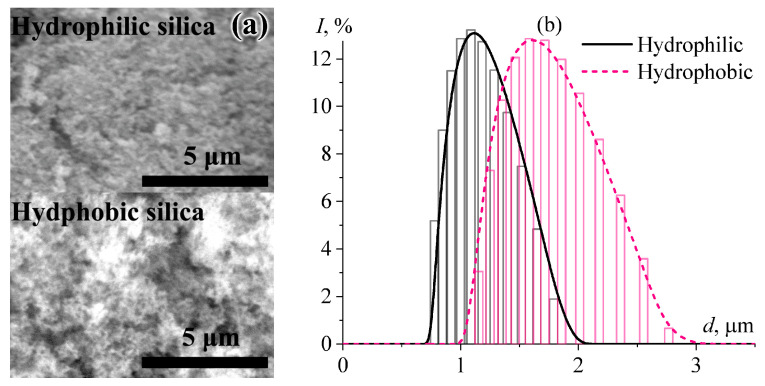
SEM images of hydrophilic and hydrophobic silica particles (**a**) and their distribution by light scattering intensity after dispersion in epoxy resin (**b**).

According to SEM images, the initial hydrophobic and hydrophilic silica nanoparticles form relatively large agglomerates (Figure 1a). The surface layer of the hydrophobic particles is more inhomogeneous, probably due to their greater tendency to agglomerate. Dynamic light scattering of silica dispersions in epoxy medium confirms these two observations. Regardless of the particles’ surface nature, they have an unimodal distribution (Figure 1b). However, according to the maxima of the curves, the average particle diameters are 1.12 μm and 1.60 μm for hydrophilic and hydrophobic particles, respectively. These sizes do not correspond to the primary particle sizes (7 nm and 12–16 nm for hydrophilic and hydrophobic silica, respectively, according to their manufacturers), indicating their agglomerated state. At the same time, hydrophobic particles are more prone to merge in the epoxy medium according to the larger sizes of their agglomerates.

Presumably, the merging of hydrophobic particles happens due to their non-polar surfaces and the relatively high polarity of the epoxy medium, which is more conducive to their interaction with each other than with the resin molecules [68,69,70]. Hydrophilic particles have polar surfaces and thus disperse better in the epoxy medium. On the one hand, the smaller the particle size of a filler, the larger the area of its contact with a continuous medium, and, thus, the more interfacial layer with modified physical properties is formed. On the other hand, the more the particles tend to agglomerate, the lower their concentration when they create a spatial gel network with a yield stress behavior in an uncured mixture. The latter effect is positive because the lower the concentration of SiO_2_ in the resin, the higher the concentration of Wood’s metal particles can be in the silica/epoxy mixture, and the higher the potential efficiency of the resultant phase-change material.

### 2.2. Rheological Properties of Silica Dispersions in Epoxy Resin

**Figure 2:**
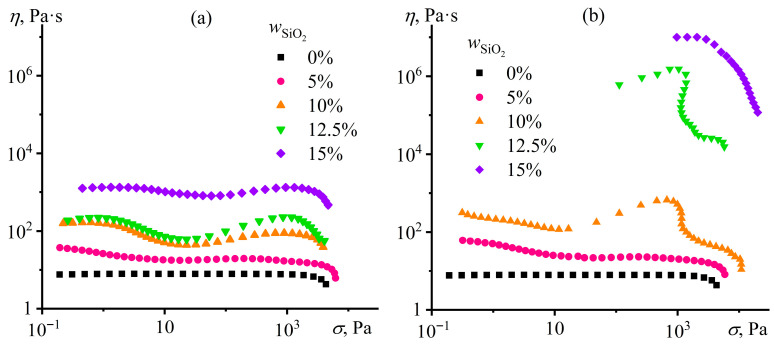
Shear stress dependencies of viscosity for epoxy resin containing silica nanoparticles with hydrophobic (**a**) or hydrophilic (**b**) surfaces at 25 °C. Legends indicate silica mass fractions.

At 25 °C, as at the elevated temperatures required to emulsify the molten metal, the neat epoxy resin exhibits Newtonian behavior and lacks yield stress, resulting in coalescence, aggregation, and sedimentation of the dispersed droplets. Silica addition may result in yield stress behavior for the immobilization of molten metal droplets. However, there is no notable yield stress even at high particle content in the case of hydrophobic silica (Figure 2a), although the viscosity gradually rises to 100 times with increasing SiO_2_ concentration up to 15%. The systems behave as non-Newtonian fluids, probably because of the agglomerated state of the nanoparticles. The viscosity exhibits a three-stage response to increasing shear rate: an initial decrease due to agglomerate breakdown [71], a slight rise from strengthened nanoparticle interactions [72], and a final drop caused by the mechanical glass transition of the epoxy medium [73].

In the case of hydrophilic silica, the agglomerate formation also occurs, according to the non-Newtonian behavior of the resultant dispersions, but only at SiO_2_ contents between 5% and 10%. At the higher silica concentrations of 12.5% or 15%, a percolating network of solid nanoparticles forms, which is manifested in gel-like rheology characterized by a pronounced yield stress and a sharp rise in effective viscosity (Figure 2b). Perhaps an appearance of yield stress is due to stronger contact bonds of hydrophilic particles interacting with each other through hydroxyl groups and hydrogen bonds [74,75]. In the case of hydrophobic particles, methyl groups coat their surfaces, which, as a result, mutually interact via weaker dispersion contacts [44].

**Figure 3:**
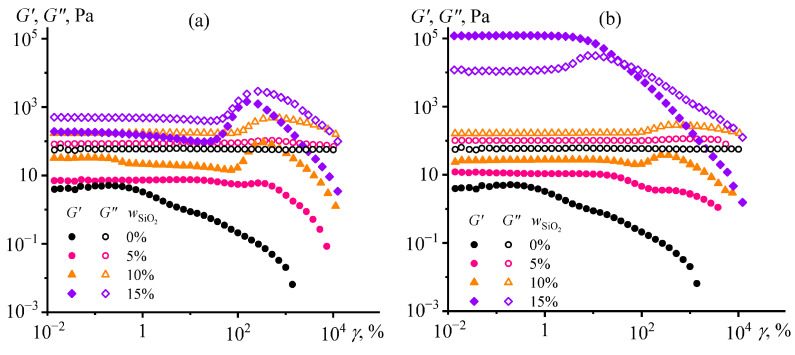
Dependencies of storage and loss moduli on strain amplitude for epoxy resin containing silica nanoparticles with hydrophobic (**a**) or hydrophilic (**b**) surfaces at 25 °C. The legends indicate silica mass fractions.

When investigating viscoelastic properties, a similar structuring pattern occurs for only one type of silica nanoparticles. In the case of hydrophobic silica, all systems behave as fluids since *G*″ > *G*′ over the entire range of strain amplitudes (Figure 3a). Solid-like behavior (when *G*′ > *G*″) is observed only at high concentrations of hydrophilic silica but in a limited range of strains up to 10–30% (*w*_SiO2_ = 15%, Figure 3b), indicating the formation of a physical gel. Higher strains decrease both moduli because of the breakdown of the silica particle network with a transition to a liquid-like behavior. However, the loss modulus passes through a maximum at *γ* = 8% before the network breakdown for the system containing 15% hydrophilic silica, which usually occurs because of shear-induce strain hardening of the system’s structure before its destruction [76]. More interestingly, the storage and loss moduli exhibit maxima for all liquid-like systems at strains of approximately 150–370%, with the peak position shifting toward lower values as the silica content increases. At these deformations, there is possibly an intensification of hydrodynamic interactions between dispersed particles, as the higher particle concentration, the higher the moduli maxima that happen at the lower strain amplitudes. More specifically, the growth of dynamic moduli may result from the shear-induced formation of fluctuating agglomerates from pyrogenic silica particles due to their branched chain-like structure [77,78]. The decrease of moduli at higher strains may be related to the destruction of fluctuating agglomerates due to the emergence of secondary flow streams or the growth of shear stress that exceeds their mechanical strength.

**Figure 4:**
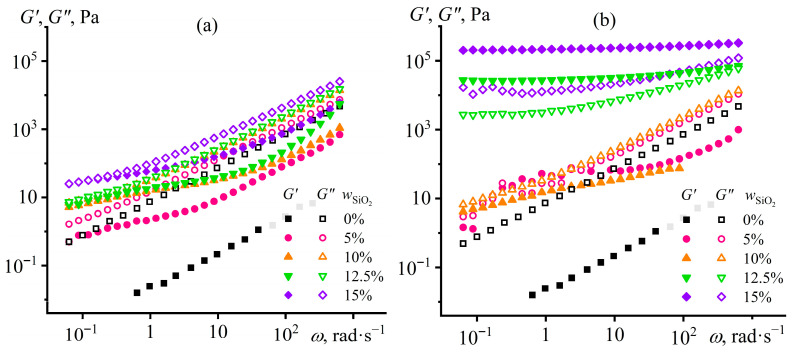
Dependencies of storage and loss moduli on angular frequency for epoxy resin containing silica nanoparticles with hydrophobic (**a**) or hydrophilic (**b**) surfaces at 25 °C. The legend indicates the mass fractions of nanoparticles.

The dependencies of the storage and loss moduli on the strain frequency confirm the existence of a gel network from hydrophilic silica particles (Figure 4). Hydrophobic particles do not form a network structure since the loss modulus exceeds the storage modulus at all considered particle concentrations and over the entire frequency range (Figure 4a). Structuring occurs only with hydrophilic nanoparticles in their 12.5% and 15% dispersions: the storage modulus becomes independent of frequency and significantly exceeds the loss modulus (Figure 4b). However, the growth of yield stress and stiffness of silica particle dispersions with increasing particle content would raise their effective viscosity. In turn, the higher viscosity would elevate the energy consumption for the emulsification of Wood’s metal in silica-containing epoxy medium until their mechanical mixing becomes impossible. Therefore, it is necessary to select the silica particle content to give the epoxy resin a low yield stress, which will nevertheless be large enough to immobilize Wood’s metal droplets.

The stability of Wood’s metal emulsions in epoxy resin requires a preliminary estimation of the specific yield stress value to prevent the coalescence and sedimentation of molten metal droplets. The pressure with which the metal droplets influence the underlying continuous medium due to gravity determines the value of the required yield stress:

(2) $$\sigma_{p}=\frac{1}{3}d\left(\rho_{d}-\rho_{m}\right)g,$$

where *d* is the diameter of metal droplets; *ρ*_d_ and *ρ*_m_ are the densities of Wood’s metal and epoxy resin, respectively; and *g* is the gravitational acceleration [79,80]. The density of the epoxy resin is 1.17 g/cm^3^ [81], whereas the density of Wood’s metal is 8.1 g/cm^3^ [82]. In addition, the ability of highly filled dispersions to flow under moderately high applied stresses is essential for their molding into the final products, as shear stresses higher than 100 Pa can break down the forming materials that lose their continuity instead of gaining fluidity [83]. Thus, it is possible to calculate the minimum yield stress required to impart stability to Wood’s metal droplets of different diameters. In our case, *σ*_p_ ≈ 2.3 Pa for the immobilization of 100 μm diameter droplets, whereas *σ*_p_ ≈ 23 Pa for larger droplets of 1 mm. At the same time, the dispersion containing 12.5% hydrophilic silica has a yield stress of about 1400 Pa (Figure 2b). Yield stress of such magnitude is sufficient to impart stability to metal droplets of giant diameter.

Thus, only hydrophilic particles with high concentration (≥12.5%) form a structural gel network capable of potentially preventing sedimentation of suspended metal particles, at least when *T* = 25 °C. However, it is also essential that the silica particles retain their structural network at high temperatures applied for dispersing the molten metal. In such a case, a phase-change material’s precursor in the form of an emulsion potentially allows for achieving a higher volume content of a phase-change agent, compared to dispersing it as a suspension. In this case, silica particles should provide dispersed metal droplets resistance to coalescence, in addition to the resistance to sedimentation. Meanwhile, the structure of hydrophilic silica in the epoxy medium may collapse upon heating because of the weakening of interparticle hydrogen bonds, just like the structuring of hydrophobic particles may conversely happen at temperature elevation. Since 12.5% silica particles are sufficient to form a structural network, it is rational to choose this concentration for further investigating hydrophilic and hydrophobic silica dispersions at higher temperatures.

### 2.3. Rheological Properties of Silica Dispersions at Heating

**Figure 5:**
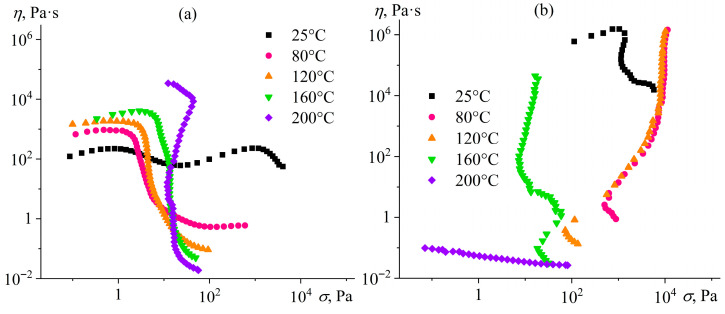
Dependencies of viscosity on shear stress for epoxy resin containing 12.5 wt% silica nanoparticles with hydrophobic (**a**) and hydrophilic (**b**) surfaces at various temperatures indicated in the legends.

Figure 5 shows the changes in effective viscosity curves upon heating an epoxy resin containing 12.5% hydrophobic or hydrophilic silica. At 25 °C, the system with hydrophobic particles does not show a pronounced yield stress (Figure 5a). However, the yield stress becomes distinctly evident as the temperature increases, probably due to a sharp drop in the epoxy resin viscosity. The lower viscosity of the epoxy continuous medium raises the difference between the highest and lowest Newtonian viscosities of silica dispersions, i.e., between the viscosities in the initial intact and completely broken states of their structures. This behavior indicates that the yield stress has a masked character at 25 °C, as the resulting viscosity decline due to the destruction of the silica particle network is slight at this temperature. In addition, the highest Newtonian viscosity and yield stress increase with rising temperature, possibly because of the enhanced dispersion interactions between hydrophobic particles or their higher interparticle contact density due to the accelerated Brownian motion during the gel network formation [84].

If the system with hydrophilic particles undergoes heating, the effective viscosity and yield stress increase initially but decrease when *T* > 120 °C. The higher yield stress may result from the strengthening of the particle gel network due to the increased interparticle contact density caused by the acceleration of particle diffusion [85], while its drop may be due to the weakening of interparticle hydrogen bonds [86,87]. At 200 °C, the yield stress disappears, although the observed shear-thinning behavior indicates the presence of particle agglomerates (not forming a structural network) and their breakdown at increasing shear stresses. Thus, a temperature of 120 °C gives the hydrophilic particle dispersion a high yield stress and is sufficient to liquify Wood’s metal, melting at 70–72 °C. Moreover, the given yield stress is higher than for hydrophobic particles of the same concentration (8000 Pa versus 4.5 Pa at 120 °C), indicating a better ability of hydrophilic silica to act as a gelator—a stabilizing agent for the dispersed Wood’s metal.

**Figure 6:**
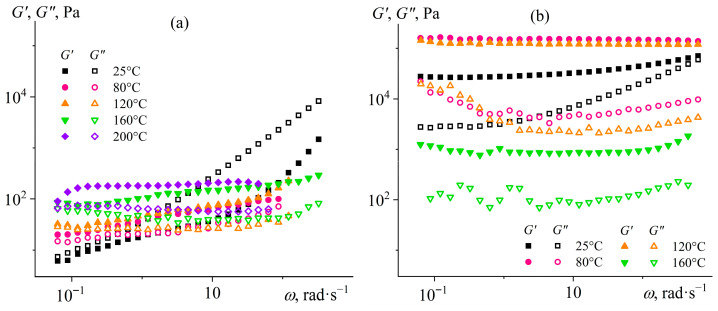
Dependencies of storage and loss moduli on angular frequency for epoxy resin containing 12.5 wt% silica nanoparticles with hydrophobic (**a**) or hydrophilic (**b**) surfaces. Legends show test temperatures. Data for hydrophilic particle dispersion at 200 °C are absent because of its inelasticity.

The frequency dependencies of the viscoelastic properties of silica dispersions are consistent with the changes in their viscosities upon heating. The system with 12.5% hydrophobic particles does not have a network structure at 25 °C since the loss modulus exceeds the storage modulus over the entire angular frequency range (Figure 6a). However, when the temperature increases, this non-structured dispersion goes to a structured state, as *G*′ > *G*″. Furthermore, the higher the temperature, the higher the dynamic moduli, i.e., the particle structure acquires a greater stiffness. In turn, the epoxy resin containing hydrophilic particles shows solid-like behavior at a lower temperature range of 25–160 °C (Figure 6b). The increase in temperature from 25 to 80 and then to 120 °C elevates the dynamic moduli, indicating an enhancement of structure formation. In contrast, higher temperatures decrease the moduli up to the disappearance of viscoelasticity at 200 °C, resulting from the disintegration of the gel network from hydrophilic particles.

**Figure 7:**
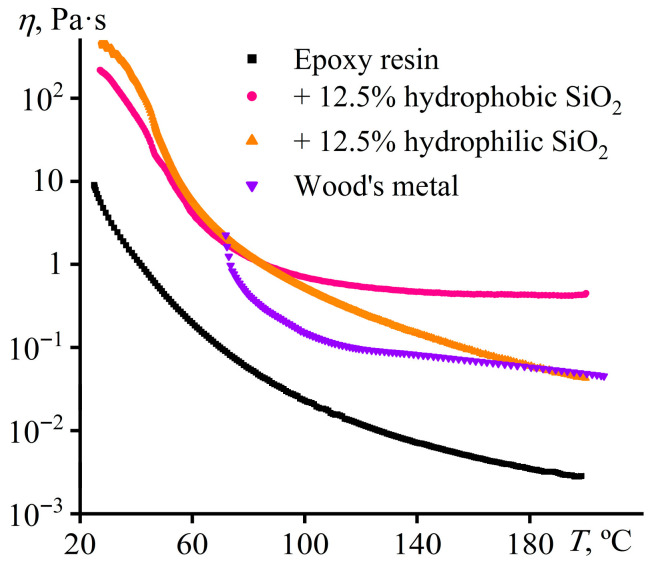
Temperature dependencies of viscosity for Wood’s metal, pure epoxy resin, and resins containing 12.5 wt% hydrophobic or hydrophilic silica at a shear rate of 10 s^−1^.

Since dispersing is more rational at a temperature higher than the melting point of Wood’s metal, it is necessary to evaluate the viscosities of the molten metal and epoxy resin, which may significantly complicate the emulsion formation, given their high difference. For effective emulsification, the components’ viscosities should be as close to each other as possible to minimize the critical capillary number, i.e., to produce smaller droplets at a lower stirring speed according to Equation (1). The viscosity–temperature dependencies for Wood’s metal, epoxy resin, and two resins containing 12.5% silica with hydrophobic or hydrophilic surfaces can help to determine the optimum dispersing temperature for the molten metal (Figure 7).

The viscosity of epoxy resin gradually decreases with increasing temperature (Figure 7). At the same time, the addition of 12.5% hydrophilic particles raises the resin viscosity by 20 times without qualitatively changing its temperature dependence. In contrast, hydrophobic silica increases viscosity at high temperatures more significantly but acts similarly to hydrophilic silica at low temperatures. Regarding Wood’s metal, its viscosity decreases sharply in the temperature region of 72–100 °C, probably due to its gradual melting (typically in the range of 60.5–75 °C [88]), and then depends less significantly on temperature, similar to the epoxy resin containing hydrophobic particles. As a result, there are two temperature regions where the viscosities of the molten metal and epoxy systems coincide to minimize the critical capillary number and reduce the size of forming droplets. The first region was observed around 72 °C, where Wood’s metal melts. However, this temperature is not convenient for dispersing the metal because of its slow melting and easy crystallizing upon the slightest cooling. The second temperature region is at 180–190 °C, where the viscosity of the molten metal is closest to that of the epoxy resin containing 12.5% hydrophilic particles. In the latter case, hydrophilic particles give the epoxy resin a higher yield stress at low temperatures than hydrophobic particles (5700 Pa versus 1 Pa at 25 °C, Figure 5), i.e., when the sedimentation stability of the epoxy system during its curing is essential. The proximity of viscosities and the higher yield stress make hydrophilic particles preferable for stabilizing Wood’s metal dispersions.

### 2.4. Rheological Properties of Epoxy Resin Containing Hydrophilic Silica and Wood’s Metal

**Figure 8:**
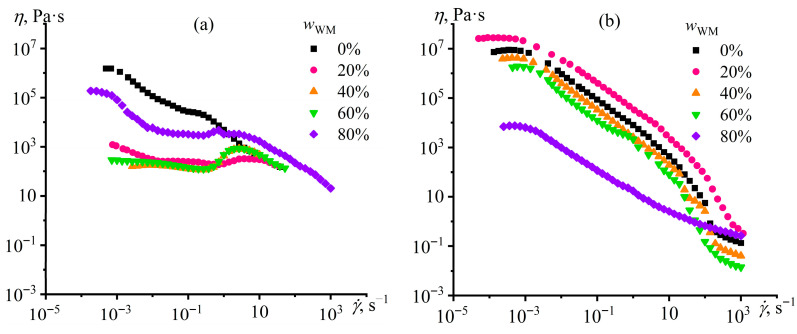

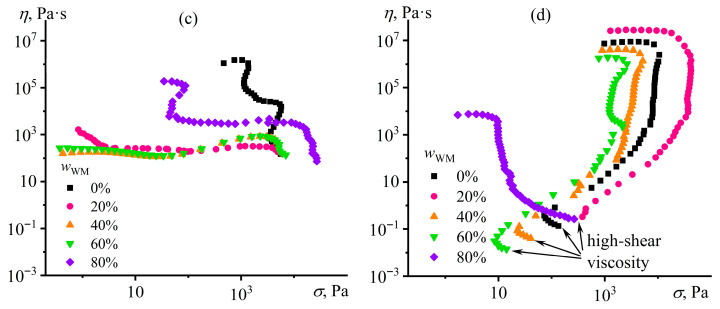
Dependencies of effective viscosity on shear rate (**a**,**b**) or shear stress (**c**,**d**) for epoxy resin containing 12.5 wt% hydrophilic silica and different mass fractions of Wood’s metal at 25 °C (**a**,**c**) and 120 °C (**b**,**d**). Legends indicate the mass fractions of Wood’s metal. The arrows show the high-shear viscosity of dispersions with ultimate broken structures.

The base silica-containing epoxy composition exhibits shear-thinning behavior at 25 °C (Figure 8a) and 120 °C (Figure 8b) due to the structuring of dispersed particles. Metal particles added to this composition decrease its viscosity at low shear rates, indirectly indicating reduced silica content within the epoxy medium. At the same time, shear-thickening behavior appears at high shear rates, probably due to the mutual friction of metallic particles under these conditions. Heating up to 120 °C increases the effective viscosity and magnifies the non-Newtonian behavior of metal-containing dispersions, indicating a stronger interaction of molten metal droplets with each other compared to their interaction in the solid dispersed state. The dependencies of viscosity on shear stress can provide more detailed information on the structuring of the systems, allowing for the evident identification of the yield stress as a narrow region of shear stresses where the effective viscosity drops vertically down.

The base composition not containing Wood’s metal exhibits yield stress at 25 °C (Figure 8c) thanks to a structural network from hydrophilic silica particles. The addition of 20% Wood’s metal leads to a sharp decrease in yield stress (from 1200 to 1 Pa) and effective viscosity, which may be because of the transfer of silica particles from the epoxy medium into the metal phase or on the metal/epoxy interface boundary. In the case of 40% and 60% Wood’s metal, the yield stress completely disappears, signifying the vanishing of the gel network from silica particles, probably due to the reduction of their concentration in the epoxy medium. Nevertheless, 80% Wood’s metal leads to the recovery of yield stress and increased viscosity, which indicates that metal particles create their structural network. Thus, the silica particles distribute themselves among the epoxy medium, the Wood’s metal particles, and their interfacial boundary, reducing their content in the epoxy resin in the presence of Wood’s metal.

Heating from 25 °C to 120 °C (Figure 8d) sharply increases the yield stress of dispersions containing up to 60% Wood’s metal, possibly due to a change in the interparticle interaction energy or/and redistribution of silica particles between the epoxy and metal phases. The viscosity profiles of dispersions containing up to 60% Wood’s metal exhibit distinctive non-Newtonian behavior. At very low shear rates, the viscosity remains high and nearly constant, indicating that the material flows while largely preserving its internal structure. This occurs when the rate of interparticle bond reformation balances or exceeds the rate of their destruction under shear [89,90]. As the shear rate increases, the shear stress rises until it reaches a critical value—the static yield stress (*σ*_s_). At this point, the percolated network undergoes irreversible breakdown, leading to a sharp drop in viscosity. With a further increase in shear rate, the shear stress itself decreases, marking the transition to a fully fluidized, shear-thinning state. This sequence produces the characteristic Z-shaped profile in the viscosity–stress curves (Figure 8d). The minimum stress on the descending branch of this “Z” corresponds to the dynamic yield stress (*σ*_d_)—the stress required to maintain the system in its broken, fluidized state. If the applied stress falls below *σ*_d_, the internal structure begins to recover. Consequently, the Z-shaped curve signifies that the stress needed to initially break the structure (*σ*_s_) is higher than the stress needed to keep it broken (*σ*_d_) [91,92]. Finally, at high shear rates, the viscosity plateaus (as indicated by arrows in Figure 8d), corresponding to a state where the dispersion structure is maximally broken down, and the flow is dominated by hydrodynamic interactions.

Throughout this study, all reported yield stress values correspond to the static yield stress (*σ*_Y_ ≡ *σ*_s_), as it determines the critical threshold between solid-like behavior at rest (preventing sedimentation of dense metal droplets) and fluid-like behavior under shear (enabling mixing and mold filling). Mastering this balance is essential for the practical processing of highly filled, uncured composites before thermal curing sets the final shape.

At 20% Wood’s metal, the yield stress increases compared with the metal-free system, from 10,600 Pa to 42,000 Pa, probably because of a rise in the quantity of dispersed phase. A further increase in the Wood’s metal concentration to 40%, 60%, and 80% gradually decreases the yield stress to 5200 Pa, 2600 Pa, and 10 Pa, respectively, consequently reducing the effective viscosity. Thus, the yield stress decrease is particularly evident in the case of 80% metal content, which may be because of the low viscosity of the molten metal droplets occupying an increasingly larger volume and reducing the overall viscosity of the resulting dispersion. Moreover, it cannot be ruled out that the observed viscosity reduction is apparent and stems from interfacial slip [93,94] at the boundary between the molten metal and the epoxy matrix.

The viscous behavior of molten metal emulsions has not been previously studied, making it impossible to compare these results directly with the existing literature. However, a similar effect has been reported for emulsions of a less viscous silicone resin dispersed in a more viscous polyisobutylene matrix, which exhibited a decrease in viscosity with increasing dispersed-phase content [95]. Notably, at resin concentrations of approximately 12–17 vol%, the viscosity drop was amplified due to the onset of shear-induced interlayer slip, driven by the alignment, elongation, and coalescence of resin droplets under flow. In the present case, the mass fraction of Wood’s metal (60–80%) corresponds to a volume fraction of roughly 15–32.5%, which is comparable to the aforementioned resin content. Thus, at high Wood’s metal loadings, droplet elongation and coalescence may occur, as the silica-containing epoxy matrix exhibits high effective viscosity—resulting in a high capillary number—and possesses a yield stress that hinders droplet shape relaxation. Under these conditions, the elongated metal droplets may facilitate interfacial slip at the metal/epoxy boundary, provided that such slip is energetically more favorable than homogeneous flow of the emulsion. This mechanism could lead to an apparent reduction in viscosity, rather than a decrease in the intrinsic molecular resistance to flow.

**Figure 9:**
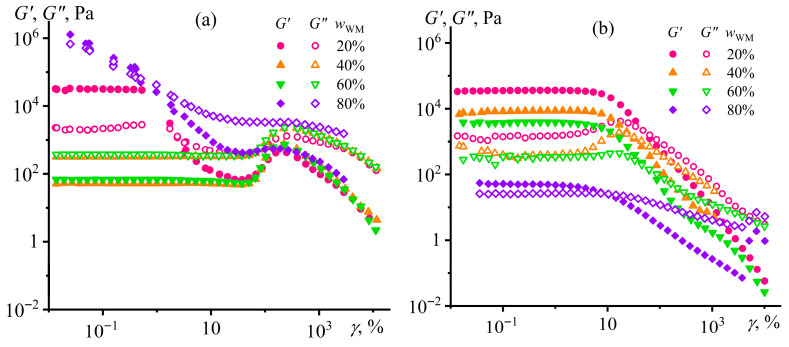
Dependencies of storage and loss moduli on strain amplitude at 25 °C (**a**) and 120 °C (**b**) for epoxy resin containing 12.5 wt% hydrophilic silica nanoparticles and different Wood’s metal mass fractions presented in the legends.

Dependencies of viscoelastic properties on strain amplitude and angular frequency may illuminate the reasons for the dispersions’ unusual behavior. At 25 °C, the systems exhibit gel-like behavior in the case of moderate (20%) or high (80%) Wood’s metal content (Figure 9a). Their storage moduli are higher than the loss moduli but only in a limited strain range of 1–2%, indicating a brittle structure that easily collapses with strain growth. In the case of 20% Wood’s metal concentration, silica particles create the gel network, whereas it is formed by Wood’s metal particles when their mass fraction is as high as 80%. At Wood’s metal concentrations of 40% and 60%, the systems have a liquid-like behavior since their loss modulus is dominant in value, which excludes the presence of a gel network. Remarkably, the amplitude dependencies of both moduli pass through local maxima at a strain of 230%, possibly due to the intensification of friction between dispersed particles with the formation of temporary spatial agglomerates and their further destruction at higher shear stresses (so-called jamming [96,97]).

At 120 °C (Figure 9b), the pattern changes slightly: there is a structural network in all systems (since *G*′ > *G*″), but it is more flexible since it withstands strains equal to 10–30% without fracture. The higher flexibility may result from the ability of molten droplets to stretch and shift relative to each other while maintaining the structural network integrity, in contrast to solid particles, whose brittle coagulation contacts break down at lower shear strains. Meanwhile, the high temperature does not change the dynamic moduli at *w*_WM_ = 20%, increases the moduli when *w*_WM_ = 40–60%, and decreases them for *w*_WM_ = 80%. The ambiguous effect of heating on the systems’ stiffness may be due to a complex set of acting factors, including redistribution of silica particles between the epoxy and metal phases, energy changes in the three types of coagulation contacts (silica/silica, metal/metal, and silica/metal), and melting of the metal phase with a decrease in its viscosity and the formation of a flexible metal/epoxy interface.

**Figure 10:**
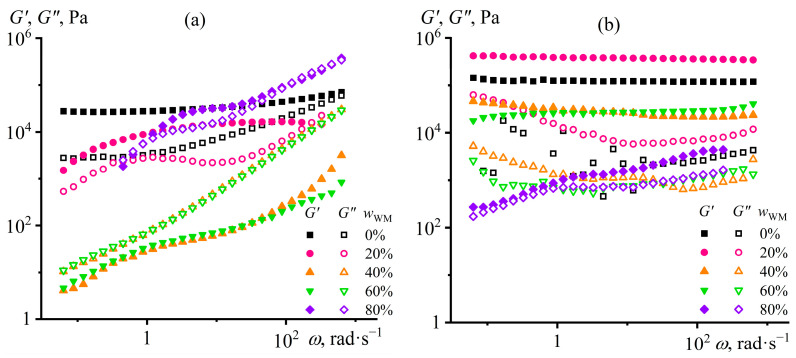
Dependencies of storage and loss moduli on the angular frequency at 25 °C (**a**) and 120 °C (**b**) for epoxy resin containing 12.5 wt% hydrophilic silica and different Wood’s metal mass fractions indicated in the legends.

The storage and loss moduli dependencies on the angular frequency confirm the different effects of Wood’s metal on the structure and rheological properties of the dispersions. At 25 °C, a gradual decrease occurs in the storage modulus when the base composition contains up to 60% Wood’s metal (Figure 10a). However, the solid-like behavior persists only at 20% Wood’s metal content in the epoxy medium, indicating its structuring. In turn, 40% and 60% metal mass fractions lead to the liquid-like behavior of the dispersions. This fact results most likely from the weakening of the gel network from silica particles due to their adsorption or absorption by the molten Wood’s metal droplets during their high-temperature emulsification. The higher the concentration of Wood’s metal, the more silica particles are extracted from the epoxy medium, and the weaker their thickening effect. At the same time, the behavior of dispersions does not obey the Maxwell model (since there is no *G*′~*ω*^2^ dependency at low frequencies), indicating their structuring, which is, however, not sufficient for gel formation. The 80% Wood’s metal causes an increase in both dynamic moduli, probably due to the formation of a new structure from metallic particles interacting with each other because of their high content. Thus, the molten Wood’s metal breaks the silica particle gel network during the high-temperature emulsification. However, the metal particles do not form coagulation contacts among themselves after they cool and crystallize, at least until 80% concentration.

Heating up to 120 °C leads to structuring (gel formation), even in metal-containing dispersions: the storage modulus is frequency-independent and exceeds the loss modulus at Wood’s metal content of 20–60% (Figure 10b). With increasing Wood’s metal content, the dynamic moduli gradually decrease compared to the base composition until gelation ceases at 80% metal concentration. The stiffness reduction and gel network destruction by Wood’s metal confirm the redistribution of silica particles between the epoxy phase, the metal phase, and their interfacial boundary compared to the base metal-free composition.

Thus, hydrophilic silica particles at moderately high concentrations immobilize dispersed Wood’s metal in solid and molten states. At low metal content, a structural network of silica particles ensures the stability of the dispersed metal phase. At the same time, a high metal content suppresses silica structure formation because of the migration of silica particles from the epoxy continuous phase on the epoxy/metal interface and/or into the metal phase. Nevertheless, the coagulation interactions of metal particles stabilize their dispersion, providing the yield stress to carry out high-temperature mixing and subsequent low-temperature curing of the metal-containing sample without settling its dispersed phase.

### 2.5. Thermophysical Properties of Cured Metal-Containing Nanocomposite Epoxy Resin

**Figure 11:**
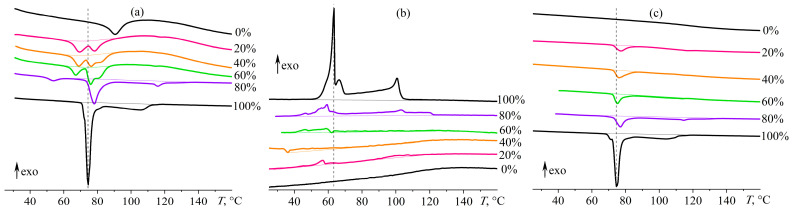
DSC thermograms obtained in first heating (**a**), subsequent cooling (**b**), and second heating (**c**) of cured epoxy compositions containing 12.5 wt% hydrophilic silica and Wood’s metal, whose mass fractions are indicated near the curves. The enthalpies of melting (Δ*H*_m_) and crystallization (Δ*H*_cr_) were determined by integrating the respective endothermic and exothermic peaks relative to the baseline heat-flow curves indicated by thinner lines; the corresponding numerical values are listed in Table 1. Vertical dashed lines mark the primary melting and crystallization transition temperatures for the pristine (100%) Wood’s metal.

According to differential scanning calorimetry (DSC) data, the glass transition temperature of the cured metal-free epoxy composition is 83 °C under heating (Figure 11a). Wood’s metal decreases the glass transition temperature, indicating a reduction in cross-link density. This effect may arise from interfacial interactions between the oxidized surface of Wood’s metal (e.g., Bi_2_O_3_, PbO) and polar components of the epoxy system—such as adsorption of epoxy or amine groups—potentially perturbing the cross-linking reaction. While such interactions could resemble acid–base or coordination effects, their exact chemical nature was not directly characterized in this study. Wood’s metal reduces the glass transition temperature by 18–20 °C when at 20–60% content, and by 34 °C at 80% content (*T*_g_, Table 1). In our case, the glass transition is accompanied by an endothermic peak (rather than a step-like change in heat flow) due to enthalpy relaxation—a phenomenon associated with physical aging of polymers stored in the glassy state [98,99]. This enthalpy relaxation occurs during the first heating of the cured samples and is absent in the second heating scan (Figure 11c). However, the step change in heat capacity at the glass transition during the second heating is poorly resolved, as the glass transition region is significantly broadened over the temperature scale. This specificity makes it difficult to reliably determine the glass transition temperature, especially for systems containing Wood’s metal, where the heat flow signal associated with the glass transition overlaps with the endothermic effect of melting the dispersed metal phase. For this reason, the glass transition temperatures reported in Table 1 were taken from the first heating scans. Note that even at high temperatures above the glass transition temperature, the epoxy matrix retains a rubbery state, thus maintaining shape stability.

After the glass transition, heated metal-containing epoxy systems undergo an endothermic transition whose temperature range expands as the mass fraction of Wood’s metal increases (Figure 11a). According to the literature data [88], Wood’s metal melts at temperatures between 60.5 and 75 °C. In our case, the initial Wood’s metal has a primary melting peak at 74.5 °C and an extended, less-pronounced melting region with a maximum of 105.5 °C. Since none of the constituent metals (Bi, Cd, Pb, and Sn) melt at these temperatures, the initial solid Wood’s metal is a heterogeneous mixture of alloys with different melting points. Therefore, the broad endothermic region indicates the alloys’ gradual melting with dissolution into the primary bulk of Wood’s metal, which transitioned to a liquid state at a lower temperature. The placing of Wood’s metal into the epoxy medium raises the melting point of its primary fraction from 74.5 °C to 76.0–78.5 °C (*T*_m_, Table 1). In addition, the thermograms evidence a second melting region with maxima at 81–82 °C (Wood’s metal content of 40–60%) or 116 °C (80%). The difference in the melting points of dispersed Wood’s metal may be due to the various sizes of its particles or the interaction of their higher-melting oxidized surfaces with the resin or hardener.

A similar pattern occurs at the subsequent cooling of the samples (Figure 11b). The thermograms reveal extended crystallization regions, especially for the initial Wood’s metal and its 80% dispersion in the epoxy medium. The Wood metal in bulk has three crystallization regions with exothermic maxima at 100.7, 66.5, and 63.5 °C, confirming the presence of hard-melting second fractions (the primary fraction’s crystallization temperatures are given in Table 1, *T*_cr_). The dispersing of Wood’s metal leads to (1) a shift of exothermic maxima by 3–4 °C towards lower or higher temperatures, (2) a decrease in the intensity of the primary crystallization peak at 56 °C, and (3) the appearance of a new crystallization region at 120 °C. In the case of less concentrated systems, the crystallization regions of the dispersed metal practically merge with the baseline because of low metal content and the crystallization’s broad temperature range. Thus, the Wood’s metal crystallizes stepwise, forming phases of different compositions with low concentrations. When heated, these phases gradually melt, dissolving into the primary fraction of Wood’s metal, which has a lower melting point than they do. In this case, the crystallization temperature of the dispersed alloy is lower than that of the bulk alloy, whereas the melting temperature is, conversely, higher for the dispersed particles. Crystallization is a kinetically controlled phenomenon that requires nucleation and crystal growth—both of which are hindered in small droplets due to the reduced probability of forming a critical nucleus. Melting, by contrast, is a thermodynamically controlled process that begins when the crystal lattice loses stability; this lattice is likely more perfect in the dispersed Wood’s metal, leading to a higher melting temperature.

Upon reheating, the melting peaks of Wood’s metal at concentrations of 20–60% become unimodal (Figure 11c). In contrast, the high-temperature shoulder observed at 80% alloy content persists, mirroring the behavior of pure Wood’s metal—indicating that this feature is intrinsic to the alloy’s melting behavior and not an artifact of its dispersion within the epoxy matrix. Moreover, the melting temperature of the dispersed alloy shifts slightly downward by 0.3–1.2 °C compared to the first heating scan, approaching the characteristic melting point of the pristine Wood’s metal. Since the droplet size could not have changed between scans and no new chemical reaction between the alloy and the cured epoxy matrix is plausible, the observed difference in melting temperature is attributed to variations in the perfection of the metallic crystals. The presence of dispersed silica nanoparticles might influence this crystalline perfection of the dispersed phase. However, the silica nanoparticles do not act as nucleating agents, as evidenced by the significantly lower crystallization temperature of the dispersed Wood’s metal in their presence compared to the bulk alloy. Thus, silica nanoparticles neither promote nucleation nor disrupt the crystal lattice. The observed increase in melting temperature may be attributed to enhanced interatomic bonding within the crystal, resulting from interactions between surface hydroxyl groups of silica and metal atoms.

In the DSC thermograms (Figure 11), melting manifests as an endothermic peak on heating (Figure 11a,c), whereas crystallization appears as an exothermic peak on cooling (Figure 11b). The corresponding transition enthalpies—obtained by integrating these peaks relative to the baseline heat-flow curves (shown as lighter lines in Figure 11)—are listed in Table 1. A comparison of the thermal effects of phase transitions reveals that the enthalpies for crystallization (Δ*H*_cr_, Table 1) and melting (Δ*H*_m_) are not equal: Δ*H*_cr_ < Δ*H*_m_, indicating thermodynamically and kinetically nonequilibrium behavior of the systems. Moreover, the stored thermal energy decreases even for pure Wood’s metal. However, the endothermic melting effect reproduces numerically upon re-heating the samples. The reproducibility of the calorimetric curves suggests that the observed reduction in the crystallization thermal effect is not genuine, likely due to an extended crystallization region caused by supercooling of the eutectic, which arises from the necessity of cooperative nucleation of multiple phases—pure metals and their intermetallic compounds. As a result, the changes in heat flow are small and invisible on the baseline background. Moreover, it cannot be ruled out that a less perfect crystalline phase forms upon cooling. In contrast, during heating, a more ideal structure (possibly recrystallized or further ordered during slow heating) is melted, resulting in a higher melting enthalpy. For these reasons, it is more appropriate to use the melting enthalpy as a more equilibrium-like quantity to calculate the relative efficiency of thermal energy storage. In this case, the nominal degree of crystallinity (DC) for Wood’s metal can be calculated as follows:

(3) $$DC=\frac{100\Delta H_{m}}{w_{WM}\times \Delta H_{m,WM}}\cdot 100\%,$$

where *w*_WM_ is the Wood’s metal mass fraction in the cured composition, and Δ*H*_m_,_WM_ is the melting enthalpy for pure Wood’s metal. It is important to emphasize that the reported degree of crystallinity is a nominal value, defined relative to the enthalpy of pristine (bulk) Wood’s metal measured under identical DSC conditions. This calculation inherently assumes that the maximum attainable melting enthalpy (Δ*H*_m_) of the metal within the composite is identical to that of the bulk alloy—an assumption that may be invalid if the emulsification or curing process alters the alloy’s microstructure (e.g., through surface oxidation or component segregation). Consequently, the DC value should be interpreted as a metric of relative enthalpy retention, not as an absolute measure of the crystalline fraction.

As calculated via Equation (3), the nominal crystallinity degree of Wood’s metal dispersed within the epoxy matrix decreases to 52–91% of its value for the bulk alloy. This reduction could be attributed to either the small size of the metal droplets (a confinement effect [100,101]) or to partial surface oxidation of the metal during the high-temperature mixing process.

An advantage of Wood’s metal as a phase-change agent is its high density, which allows it to store more energy per unit volume, Δ*H_V_*. For pure Wood’s metal, Δ*H_V_* is 243.8 J/cm^3^, whereas the Δ*H_V_* of pure paraffin wax is around 170 J/cm^3^ [102]. In the case of 80% Wood’s metal content, Δ*H_V_* is 120.8 J/cm^3^, which is comparable with the value for pure paraffin wax; however, paraffin wax cannot retain its shape at high temperatures, unlike the cured metal-containing epoxy matrix.

### 2.6. Mechanical Properties of Cured Metal-Containing Nanocomposite Epoxy Resin

For shape-stable PCMs, the critical mechanical requirement is not tensile or impact strength—as for structural composites—but dimensional integrity during phase transition, which is governed by the storage modulus and yield stress. Accordingly, dynamic mechanical analysis (DMA) was selected as the primary mechanical characterization technique, as it directly quantifies the stiffness (storage modulus, *G*′) and glass transition behavior that dictate form stability under thermal cycling. DMA of the cured composites provides detailed insight into how the inclusion of Wood’s metal affects their thermophysical and mechanical properties, specifically the evolution of the storage modulus and glass transition temperature as a function of metal loading.

**Figure 12:**
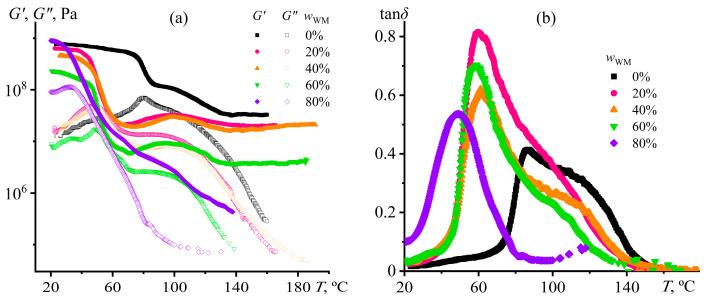
Temperature dependencies of the storage and loss moduli (**a**) and the loss tangent (**b**) for cured epoxy compositions containing 12.5 wt% hydrophilic silica and Wood’s metal, whose mass fraction is indicated in the legend.

According to DSC data (Table 1), Wood’s metal significantly reduces the glass transition temperature of the cured compositions compared to the silica-filled epoxy resin without Wood’s metal (*w*_WM_ = 0%), and thus is expected to reduce their stiffness as well. Indeed, the temperature dependencies of storage and loss moduli (Figure 12a) show a decrease in the storage modulus at 25 °C (*G*′_25°C_, Table 2) compared to the base matrix as the concentration of Wood’s metal increases to 60%. Meanwhile, the storage modulus re-increases at 80% metal concentration and exceeds the storage modulus for the initial epoxy matrix. The high content of metal particles likely has a reinforcing effect on the epoxy matrix despite their plasticizing effect expressed via the reduction in cross-linking density and decrease in *T*_g_ (Table 1). The heating decreases the storage modulus because of the loss of the glass state, which occurs at lower temperatures in the Wood’s metal presence. In addition, after the glass transition point, the curves show a slight increase in the storage modulus as a broad peak with a maximum of 100 °C, indicating a post-curing reaction. The DSC thermograms reveal a broad exothermic maximum at 100–120 °C (Figure 11a), which is thus tentatively assigned to post-curing reactions. Notably, this feature is absent in the second heating scan (Figure 11c), confirming its irreversibility.

Thus, Wood’s metal reduces the active concentration of either the hardener or the epoxy resin in the total volume of the reaction system, resulting in a lower curing degree—lower *T*_g_ and *G*′. Conceivably, the molten Wood’s metal droplets adsorb some hardener or resin molecules that become unavailable for the curing reaction. In addition, the decrease in the effective concentration of reactive groups may be influenced by interfacial interactions between the oxidized metal surface and the epoxy/hardener system. While such interactions could reduce the availability of amine or epoxy groups for cross-linking, this remains a plausible hypothesis in the absence of direct spectroscopic evidence.

The reduction in the rubbery-plateau modulus (*G*_N_^0^, i.e., *G*′ at about 160 °C, where *G*′ ≈ *const*) confirms the decrease in the cross-link density, which is less the more Wood’s metal was added. The cross-link density *q* is related to the rubbery-plateau modulus as follows [102,103]:

(4) $$q=\frac{\rho k_{B}TN_{A}M_{m}}{G_{N}^{0}}$$

where *ρ* is the cured polymer density, *N*_A_ is the Avogadro constant, *T* is thermodynamic temperature, *k*_B_ is the Boltzmann constant, and *M*_m_ is the monomer molecular weight. In our case, *G*_N_^0^ drops from 32.8 MPa to 19.9 MPa, 18.2 MPa, and 3.9 MPa at 20%, 40%, and 60% Wood’s metal content, respectively (Table 2). In turn, this fact indicates a corresponding decrease in the cross-link density to 61%, 55%, and 12% from the initial nominal 100% value. In the case of 80% metal content, the modulus falls even more dramatically, indicating a drop in the cross-link density by at least 76 times (to about 1% of its initial value). It should, however, be noted that the rubbery plateau modulus in this case is determined with considerable uncertainty, as its value is influenced not only by cross-links in the epoxy polymer network but also by the dispersed metal droplets.

According to the temperature dependency of the loss tangent, the metal-free epoxy composition has a broad glass transition region from 70 to 140 °C (Figure 12b). In this case, a maximum loss tangent occurs at 86 °C. Still, there is also a high-temperature shoulder, possibly caused by the adsorption of epoxy chains on the silica surfaces with decreasing segmental mobility. As the Wood’s metal concentration increases, the glass transition region shifts towards lower temperatures from 86 °C to 49 °C (*T*_g,tan*δ*_, Table 2), confirming the reduction in the cross-linking degree. Meanwhile, the high-temperature shoulder gradually disappears, which may be because of a decrease in the silica concentration within the epoxy medium due to the transition of silica nanoparticles to the metallic phase or on its interfacial boundary. As a result, the fraction of adsorbed epoxy chains decreases, leading to the disappearance of the high-temperature shoulder.

### 2.7. Morphology of Cured Metal-Containing Nanocomposite Epoxy Resin

**Figure 13:**
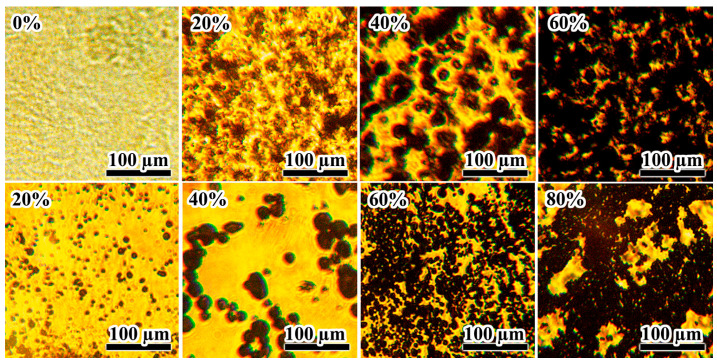
Microphotographs of uncured epoxy compositions containing 12.5 wt% hydrophilic silica and different concentrations of Wood’s metal particles, whose mass fraction is indicated in the top row. The bottom row shows the same compositions after their 5-time dilution with acetone.

The morphology studies of metal-containing epoxy dispersions allow for assessing their structure before and after curing. The microphotograph of the uncured metal-free epoxy composition clearly shows its optical inhomogeneity caused by the silica nanoparticle agglomerates (Figure 13, 0%). When the epoxy composition contains Wood’s metal, agglomerates of silica nanoparticles mix with metal particles, causing the spatial structural network to become black. As the concentration of Wood’s metal increases (Figure 13, top row), the structural network becomes denser until nearly light-tight.

The dilution of the same uncured samples with acetone allows for a better view of the metal particles (Figure 13, bottom row). At the initial Wood’s metal content of 20%, the particle agglomerates disappear because of dilution, i.e., there was no coalescence of metal droplets with the formation of phase contacts between them during emulsification of the molten metal. At Wood’s metal content of 40%, both individual metal particles and their chain agglomerates are present. In this case, the particle size increases, possibly because of the coalescence of molten metal droplets during their emulsification. Wood’s metal mass fractions of 60% and 80% lead to the formation of reticulate spatial agglomerates with a decrease in the primary droplet size and an increase in the particle network density when going from 60% to 80% of the metal content. Thus, the metal particles form agglomerates and a gel network (even in dilute dispersions) as their content increases. However, it is unclear whether phase contacts occur in the concentrated metal dispersions or network formation happens only because of the particle coagulation.

**Figure 14:**
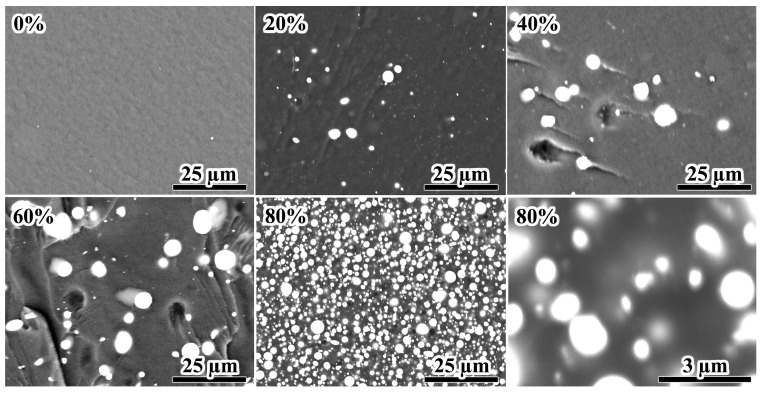
Cross-section SEM images of cured epoxy compositions containing 12.5 wt% hydrophilic silica and different concentrations of Wood’s metal, whose mass fraction is indicated in the upper left corner.

Scanning electron microscopy allows for studying the shape and size of Wood’s metal particles and their aggregates in more detail (Figure 14). In all cases, the metal particles have a round shape because of their formation via emulsification. In addition, no particle aggregates are present even at 80% metal content. An elevation of the Wood’s metal content from 20% to 80% first increases the average metal particle diameter from 2.4 μm to 5.3 μm and then decreases it to 1.7 μm (Table 3). On the one hand, the increase in the size of droplets may be due to their coalescence during the emulsification. However, there is no merging of droplets according to the SEM image of even the most concentrated system at its high magnification. Moreover, some metal particles are as small as 0.1 µm in diameter at their 80% content. On the other hand, the variation in the composition of the epoxy system by adding Wood’s metal changes its viscosity. As a result, the capillary number achieved during metal emulsification under the same, equal conditions also changes, consequently altering the final droplet size. Emulsification occurs at high shear rates when the gel network from silica particles completely breaks down, minimizing the dispersion viscosity. In this case, the change of the Wood’s metal content from 20% to 40% and then to 60% reduces the high-shear viscosity of the epoxy composition under mix (Figure 8b, *η* at about 1000 s^−1^). In turn, this effect proportionally increases the radius of the formed droplets according to Equation (1). However, a rise in the Wood’s metal concentration up to 80% dramatically elevates the high-shear viscosity because of a reduction in the formed-droplet size. Thus, the epoxy compositions’ rheological properties determine the sizes of the formed droplets and their resistance to coalescence via the high-shear viscosity and the emergence of the yield stress behavior, respectively.

**Figure 15:**
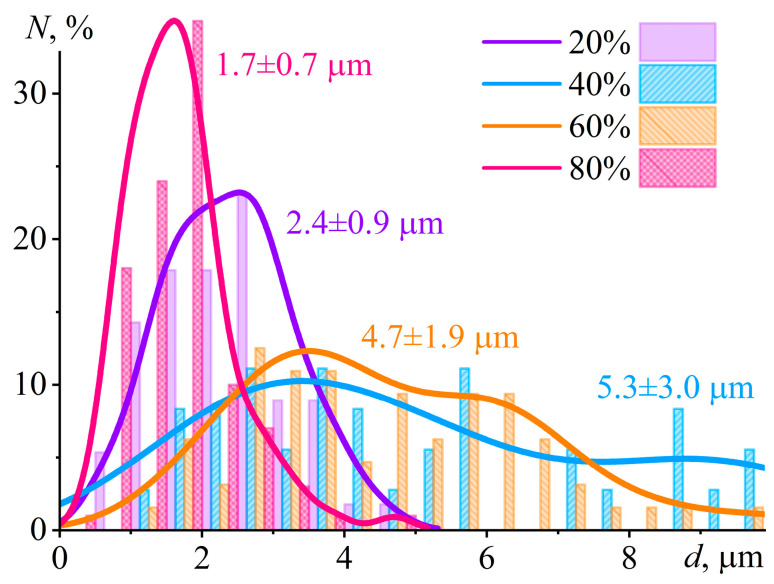
Particle size distribution of Wood’s metal droplets in cured epoxy composites, as determined by quantitative SEM image analysis. The histograms display the experimental frequency distribution of droplet diameters. The smoothed curves overlaid on the histograms are kernel density estimates added to guide the eye. The legend specifies the mass fraction of Wood’s metal in each composite. The mean particle diameters are indicated near the curves.

The particle size distributions derived from SEM image analysis (Figure 15) demonstrate a pronounced influence of metal loading on droplet coalescence. Composites containing 20% and 80% Wood’s metal exhibit narrow, unimodal distributions with mode diameters of 2.5 µm and 1.6 µm, respectively, indicative of effective suppression of coalescence. In stark contrast, systems with intermediate loadings (40% and 60%) display broad, distinctly bimodal distributions. The appearance of secondary modes at approximately 9 µm (40%) and 6 µm (60%) provides direct morphological evidence of significant droplet coalescence during the cooling and solidification stage. This trend correlates directly with the yield stress measured at 25 °C (Figure 8c). Only the 20% and 80% formulations retain a finite yield stress upon cooling to room temperature—sufficient to immobilize metal droplets and prevent coalescence. Conversely, the 40–60% systems lack a percolated network capable of imparting yield stress at 25 °C, allowing for droplet rearrangement and merging. These findings underscore that the preservation of a finite yield stress—whether originating from a bulk silica network or an interfacial layer—during the critical cooling phase is essential for long-term morphological stability. Notably, coalescence is effectively suppressed even at the high loading of 80%, suggesting that silica nanoparticles adsorbed at the metal/epoxy interface provide not only steric repulsion but also significant mechanical resistance against film drainage and droplet coalescence.

It should also be noted that high-magnification SEM imaging of the 80% system reveals faint, asymmetric bright halos (0.1–1 µm wide) surrounding the metal particles (Figure 14, bottom right). The irregular shape of these halos is consistent with an island-like coverage of the metal droplets by silica aggregates. The average size of these aggregates in epoxy resin is 1.12 μm (as determined by light scattering; Figure 1b) and corresponds to the dimensions of the widest observed halos. Although similar features are not readily apparent at lower metal contents (20–60%), their absence could be attributed to the reduced contrast of adsorbed silica aggregates against the silica-rich epoxy matrix. At 80% Wood’s metal, the metal droplets likely adsorb a major portion of the dispersed silica particles from the epoxy matrix. This adsorption would deplete silica from the continuous phase and concentrate it at the interface, thereby enhancing the contrast between the epoxy matrix and the adsorbed silica aggregates. Notably, the detection of these halos aligns with the rheological evidence for silica redistribution between the epoxy continuous medium and the metal/epoxy interface. Thus, the observed halos provide consistent morphological support for the proposed mechanism of interfacial stabilization, in which adsorbed silica nanoparticles are indispensable for preventing coalescence of molten metal droplets. Without hydrophilic silica, the system would undergo immediate coalescence and sedimentation, as confirmed by the absence of stable molten metal emulsions in silica-free systems.

### 2.8. Stabilization Mechanism of Wood’s Metal Droplets

The rheological and morphological data reveal that the stabilization of Wood’s metal in an epoxy matrix is not governed by a single mechanism but depends on both metal loading and temperature. At 25 °C and in the absence of metal, 12.5% hydrophilic silica forms a percolating network that imparts a yield stress of about 1400 Pa (Figure 2b). This physical gel immobilizes solid particles and prevents their sedimentation during low-temperature curing.

Upon addition of molten Wood’s metal and heating to 120 °C, the yield stress of the same system drops sharply—even vanishing at 40–60% metal (Figure 8c)—while the droplets remain spherical and show only slight signs of coalescence (Figure 14 and Figure 15). The simultaneous loss of bulk yield stress and retention of droplet integrity suggests that silica nanoparticles migrate from the epoxy bulk to the metal/epoxy interface, where they may function analogously to Pickering stabilizers despite their native hydrophilicity and absence of surface modification. This redistribution is further supported by a sharp reduction in low-shear viscosity (Figure 8a) and the disappearance of solid-like viscoelasticity (Figure 10a).

At 80% metal, the system regains a finite yield stress (*σ*_Y_ ≈ 100 Pa at 25 °C), viscosity rises (Figure 8a), and *G*′ exceeds *G*″ at low strains (Figure 9a). Crucially, SEM shows no droplet coalescence (Figure 14 and Figure 15), while dilution experiments reveal a reticulated arrangement of ~1.7 µm droplets (Figure 13, Table 3). Given that Wood’s metal is solid at 25 °C, the observed yield stress likely originates from a percolated network of rigid metal particles in direct contact, stabilized by interparticle friction and possible silica bridging at contact points. Rheological data at 120 °C (Figure 8d and Figure 9b) confirm that this structure persists even during emulsification: *G*′ > *G*″ over a broad strain range, and a finite yield stress (*σ*_Y_ ≈ 10 Pa) remains—sufficient to counteract sedimentation of droplets ≤ 100 µm (*σ*_p_ ≈ 2.3 Pa, Equation (2)).

**Figure 16:**
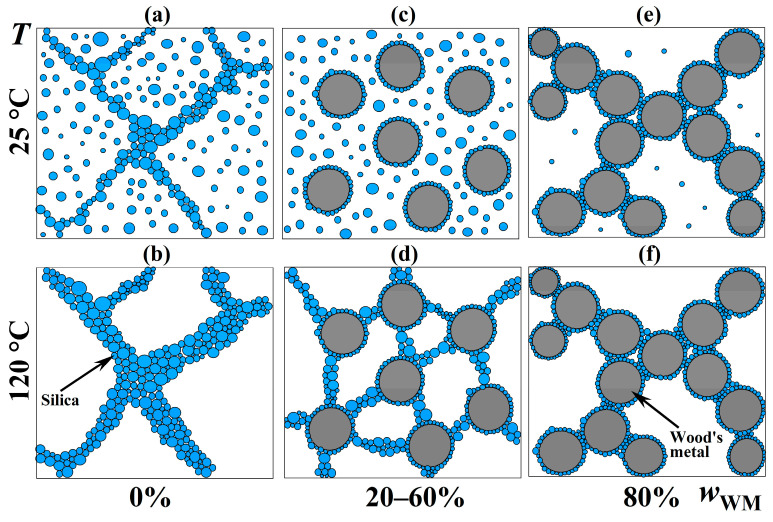
Schematic illustration of the redistribution of hydrophilic silica nanoparticles in epoxy resin/Wood’s metal systems as a function of metal loading and temperature: 0% Wood’s metal at 25 (**a**) and 120 (**b**) °C; 20–60% Wood’s metal at 25 (**c**) and 120 (**d**) °C; 80% Wood’s metal at 25 (**e**) and 120 (**f**) °C. In the absence of metal, silica forms a bulk percolating network (**a**), which is thermally reinforced upon heating (**b**). At intermediate metal loadings, silica nanoparticles partition toward the metal/epoxy interface; this migration partially preserves the gel network at 120 °C (**d**) but disrupts the bulk network upon cooling (**c**). At 80%, the metal particles themselves form a percolated assembly; this structure is stabilized by direct interparticle contacts mediated by adsorbed silica nanoparticles, resulting in a friction-stabilized network both when the metal is solid (**e**) and when it is molten (**f**).

A schematic representation of the temperature- and concentration-dependent redistribution of hydrophilic silica nanoparticles is provided in Figure 16. Figure 16a,b illustrates the metal-free system. At 25 °C, hydrophilic silica forms a percolating network with a high yield stress (*σ*_Y_ ≈ 1400 Pa). Upon heating to 120 °C, the yield stress increases significantly (*σ*_Y_ ≈ 8000 Pa), suggesting that enhanced particle mobility allows for a more complete network formation as particles overcome kinetic barriers to bonding.

Figure 16c,d corresponds to systems with 20–60% Wood’s metal. During emulsification at 120 °C, silica adsorbs at the metal/epoxy interface. This migration disrupts the bulk gel upon cooling to 25 °C (*σ*_Y_ < 1 Pa at 20% loading, vanishing entirely at 40–60%; Figure 8c) while effectively preventing significant droplet coalescence (Figure 14). At 120 °C, however, the residual silica remaining in the epoxy phase enables partial recovery of the gel network, restoring a considerable yield stress (*σ*_Y_ ≈ 2600–42,000 Pa, decreasing with metal loading; Figure 8d). This ensures the emulsion’s stability during high-temperature processing.

Figure 16e,f depicts the system with 80% metal. At 25 °C, the solidified metal particles form a percolated network. The contacts between these particles are mediated by adsorbed silica nanoparticles, giving rise to a friction-stabilized mechanical resistance (*σ*_Y_ ≈ 100 Pa; Figure 8c). At 120 °C, the molten droplets remain kinetically stable and sustain a finite yield stress (*σ*_Y_ ≈ 10 Pa; Figure 8d), the decrease of which is likely due to the weakening of interparticle silica hydrogen bonds. This behavior is consistent with interdroplet contacts that are also maintained via rigid silica layers adsorbed on droplet surfaces, as evidenced by the morphological analysis (Figure 14, bottom right).

Thus, the system exhibits three distinct stabilization regimes: from bulk gelation at low metal contents, to interfacial anchoring during emulsification, and finally to droplet-network formation at high loadings. The absence of chemical modification distinguishes this approach from classical Pickering emulsions that rely on engineered particle wettability. This also explains why hydrophobic silica—while capable of creating structure at elevated temperatures (Figure 5a)—fails to provide sufficient low-temperature stability for curing (*σ*_Y_ < 2 Pa at 25 °C). In contrast, hydrophilic silica ensures both high-temperature emulsion integrity and room-temperature immobilization. This hybrid mechanism enables metal loadings up to 80 wt%, a level previously unattained in metal-in-polymer phase-change composites.

## 3. Conclusions

The morphology and physicochemical properties of epoxy-based phase-change materials containing silica nanoparticles and a model low-melting-point alloy—Wood’s metal—were investigated both before and after curing, revealing the following key findings:

- The addition of 12.5–15 wt% hydrophilic silica nanoparticles induces physical gelation of the epoxy resin, forming a yield-stress nanocomposite gel, which significantly increases both the elasticity and the effective viscosity at 25 °C. As the temperature increases, this yield stress persists in systems with hydrophilic silica. In contrast, dispersions with hydrophobic silica develop a yield stress only upon heating, indicating the formation of a more structured system at elevated temperatures.
- Hydrophilic silica works better for immobilizing dispersed Wood’s metal in the epoxy-based medium because of the proximity in viscosities of the molten Wood’s metal and the silica/epoxy dispersion, which minimizes the critical capillary number during their mixing and, consequently, the resulting metal droplet size.
- Wood’s metal reduces the yield stress of hydrophilic particle dispersions up to its disappearance at 25 °C because particles adsorb on the metal/epoxy interface or are absorbed by molten metal droplets during high-temperature emulsification. However, Wood’s metal forms a percolated gel network at 80 wt% content, leading to the kinetic stability of its dispersion at both low and high temperatures, likely facilitated by interfacial coverage with silica nanoparticles, though direct interfacial evidence was not obtained in this study.
- At 80 wt% content, Wood’s metal decreases the glass transition temperature of cured epoxy compositions by 34 °C, indicating a reduction in cross-link density, but increases their storage modulus thanks to the reinforcing effect of solid spherical metal particles that do not form aggregates and have an average size of 2 µm.
- Wood’s metal melts and crystallizes gradually, decomposing into phases of different compositions, reflected in the extended length of phase transitions on the temperature scale. The nominal crystallinity of Wood’s metal dispersed in the epoxy matrix decreases but remains high enough to store thermal energy up to 120.8 J/cm^3^ at Wood’s metal mass fraction of 80%.

The key advantage of this work is the demonstration of a novel gel-based mechanism for stabilizing molten metal droplets using unmodified hydrophilic silica nanoparticles. This mechanism suggests a general potential for creating stable, high-loading emulsions of various low-melting-point metals within an epoxy matrix. The resulting phase-change composites exhibit form stability and high volumetric energy storage density. Importantly, this approach is not limited to the specific materials studied. It can be extended to other fusible alloys (e.g., Bi–Sn, In–Ga, or Field’s metal), provided their melting points lie within the processing window of the polymer—above its cure temperature but below its degradation temperature. Similarly, other thermosets with hydrogen-bonding capability (e.g., polyurethanes, cyanate esters, or bio-based epoxies) are expected to support the formation of an analogous stabilizing silica network. The overarching design principle emerging from this study is the critical balance between silica content, metal loading, and processing temperature. This balance governs the dynamic transition from bulk gelation to interfacial stabilization—a mechanistic framework applicable to a broad range of particle-stabilized metal-in-polymer composites. It is acknowledged that the specific Wood’s metal/epoxy system investigated here has certain limitations: the relatively high melting point of the metal phase-change agent, the broadening of its phase transition over a temperature range, and its incomplete crystallization within the cured epoxy matrix. These inherent properties of Wood’s metal define the specific performance characteristics of the composite but do not invalidate the general stabilization mechanism. In addition, although this work demonstrates the fabrication and initial performance of high-loading composites, their long-term thermal cycling stability, which is critical for practical use, requires further validation. Preliminary tests (5–10 heating–cooling cycles between 25 °C and 120 °C) across the Wood’s metal melting point (72 °C) show no macroscopic leakage, cracking, or delamination. Nevertheless, systematic studies over hundreds of cycles are needed to comprehensively assess the material’s durability, specifically in terms of latent heat retention, mechanical stability, and interfacial evolution. Another disadvantage is the presence of toxic cadmium and lead in Wood’s metal, limiting the broad use of the obtained phase-change materials. Nevertheless, these metals retain industrial utility in strictly controlled applications, most notably for radiation shielding. In such contexts, their high atomic number, density, and favorable cost-to-performance ratio are deemed critical and justify their use despite environmental concerns, especially when encapsulated within a cross-linked polymer matrix that effectively contains the risk of leaching. Indeed, owing to the high mass fraction of high-atomic-number elements (Pb, Bi, and Cd) in Wood’s metal, the developed composites could also be considered as candidate materials for X-ray and gamma-ray shielding in future investigations. They may be particularly suited for applications that require moldable, non-structural radiation barriers, such as in medical or nuclear installations. This potential application is consistent with the well-established use of heavy-metal-filled polymers for attenuating ionizing radiation. It is important to note, however, that quantitative shielding performance was not assessed within the scope of this study, which focused primarily on phase-change functionality. The flowability of the metal-containing uncured epoxy compositions allows for their molding into complex or large-scale shapes, e.g., to form walls of underground shelters or radioactive waste repositories with their subsequent curing at ambient temperature. Moreover, their phase-change behavior enables a viscosity reduction by several orders of magnitude at elevated temperatures, which facilitates complex molding but necessitates a carefully matched curing agent to prevent premature cross-linking.

Further studies should include the determination of the plasticization mechanism of the epoxy matrix by Wood’s metal, the search for an optimal non-stoichiometric hardener/epoxy ratio to increase the curing degree and glass transition temperature of the resultant materials, the investigation of their thermal conductivity, their resistance to repeated heating/cooling cycles, and their ability to shield gamma/X-ray radiation. In addition, cadmium- and lead-free low-melting-point metal alloys warrant examination as functional agents for epoxy-based phase-change materials or radiation-protective films, coatings, or structural elements, especially for sustainability-driven applications where environmental compliance and regulatory restrictions are paramount.

## 4. Materials and Methods

### 4.1. Materials

The diglycidyl ether of bisphenol A (DER-330, Dow Chemical, Midland, MI, USA), containing 180.5 g/mol-eq of epoxy groups, was used as the continuous epoxy medium. Wood’s metal (Ural Plant of Chemical Reagents, Verkhnyaya Pyshma, Russia), with a melting point of 70–72 °C, was used as the phase-change agent. According to energy-dispersive X-ray spectroscopy, its mass composition included 31.9% Cd, 38.2% Pb, 23.8% Bi, and 6.1% Sn. Silicon dioxide nanoparticles with hydrophilic or hydrophobic surfaces were used to create yield stress in the epoxy resin. Hydrophilic pyrogenic silica Aerosil 380 (Sigma Aldrich, St. Louis, MO, USA) with a primary particle size of 7 nm and a specific surface area of 388 m^2^/g was used. Hydrophobic silica Aerosil R972 (Evonik Industries AG, Essen, Germany) obtained via treating pyrogenic silica with dimethyldichlorosilane, with a primary particle size of 12–16 nm and a specific surface area of 110 m^2^/g, was used.

The content of silica nanoparticles in epoxy resin without Wood’s metal was 5, 10, 12.5, and 15 wt% (*w*_SiO2_), while the hydrophilic silica content for the blends with Wood’s metal was 12.5 wt%. The curing agent was diethylenetriamine (DETA) produced by Sigma-Aldrich with an amine content of 20.6 g/mol-eq. The resin/hardener ratio was stoichiometric (89.7/10.3 wt%/wt%). Wood’s metal fraction in the composite epoxy matrix containing 12.5 wt% hydrophilic SiO_2_ was 0, 20, 40, 60, and 80 wt% (*w*_WM_).

Epoxy resin and silica nanoparticles were mixed on a HuXi HR-25 rotor-stator disperser (Shanghai, China) with a rotor speed of 30,000 rpm at 25 °C. After that, coarse pellets (3–4 mm) of Wood’s metal were added to the obtained silica/epoxy blend, followed by heating to 190 °C to melt them and subsequent dispersion in the liquid state at the same rotor speed and a constant temperature of 190 °C. The hardener (DETA) was added last, after rapidly cooling the metal/silica/epoxy mixture to 25 °C in a 5 °C water bath, without degassing to prevent premature curing and structural changes. The samples were cured in two stages: firstly, at 25 °C for 24 h, and then at 60 °C for 1 h.

### 4.2. Methods

Dynamic light scattering on an analyzer, Litesizer 500 (Anton Paar, Graz, Austria), estimated the sizes of silica particles. An epoxy dispersion containing 5% hydrophilic or hydrophobic silica was diluted 5 times with acetone to obtain a 1% concentration. The particle size distribution in the resulting 1% dispersion was determined at 25 °C using a laser wavelength of 658 nm and a scattering angle of 175°.

Rheological properties were investigated on a rotary rheometer DHR-2 (TA Instruments, New Castle, DE, USA). Flow curves and frequency dependencies of the storage and loss moduli were obtained for uncured epoxy resins containing silica nanoparticles. Investigations were performed using a plate/plate measuring geometry with a plate diameter of 25 mm and a gap of 1 mm. Flow curves were measured under a stepwise increase in the shear rate from 10^−4^ to 1000 s^−1^ at 25, 80, 120, 160, and 200 °C. Frequency dependencies of the storage and loss moduli were obtained at the same temperatures with a strain amplitude of 0.1% and an angular frequency range of 0.0628–628 rad/s. Temperature dependencies of effective viscosity were measured at a shear rate of 10 s^−1^ and a heating rate of 5 °C/min for a temperature range of 25–200 °C. The viscous and viscoelastic properties of uncured samples containing Wood’s metal were investigated under the same conditions, except that the studies were carried out at temperatures of 25 and 120 °C using an 8 mm diameter parallel-plate geometry with a 0.5 mm gap. Rheological characteristics were calculated according to standard equations [96] with relative measurement error not more than 5%. To ensure the reliability of the data, all experiments were conducted with a minimum of two replicates to assess reproducibility.

Mechanical characterization in this study was focused on dynamic mechanical analysis in bending mode. This focus is justified because the primary performance criterion for shape-stable phase-change materials is their ability to retain dimensional integrity during thermal cycling, rather than ultimate tensile or impact strength. DMA provides direct, temperature-dependent measurements of the storage modulus (stiffness) and glass transition behavior, which are the key properties governing the form stability under service conditions. Rectangular beams 25 mm long, 12 mm wide, and 4 mm thick were used to perform the DMA of the cured samples. Measurements were done by the two-point bending method using a single-cantilever clamp on the same rotary rheometer at a frequency of 1 Hz, a relative strain amplitude of 0.01%, a heating rate of 2 °C/min, and within a temperature range from 25 °C to 200 °C. The relative error in measuring the rheological characteristics was less than 5%.

Differential scanning calorimetry of cured samples was performed in an argon atmosphere using a calorimeter DSC 214 Polyma (Netzsch, Selb, Germany). Heat flow was gauged at a heating or cooling rate of 10 °C/min within 25–180 °C. The relative error of enthalpy measurement did not exceed 5%, and the accuracy of transition temperature determination was ±0.2 °C. Each test was repeated at least twice to verify reproducibility.

Microphotographs of Wood’s metal particles after their dispersion in the silica-contained epoxy resin were obtained using a ×25 objective lens and a digital camera with a 12-megapixel 1/1.7-inch sensor Sony IMX226 (Tokyo, Japan).

The morphology of cured samples was studied using a scanning electron microscope Phenom XL G2 (Thermo Fisher Scientific, Eindhoven, The Netherlands) with an accelerating voltage of 15 kV and a pressure of 60 Pa. Sample preparation consisted of a brittle break after curing, followed by ion-plasma sputtering of silver onto the resulting fracture surfaces on an instrument 108 Auto (Cressington Scientific Instruments, Watford, UK) to a layer thickness of 5 nm. Imaging of silica nanoparticles consisted of their application to carbon adhesive tape before silver sputtering and the following investigation. Energy-dispersive X-ray spectroscopy of the Wood’s metal surface to analyze its mass fraction composition was performed on the same electron microscope.

## Figures and Tables

**Table 1:** Thermophysical characteristics of cured epoxy compositions containing 12.5% hydrophilic silica and different mass fractions of Wood’s metal.

*w*_WM_, wt%	*T*_g_, °C	*T*_cr_, °C	*T*_m_, °C *	Δ*H*_cr_, J/g	Δ*H*_m_, J/g	Δ*H_V_*, J/cm^3^	DC, %
0	83.0	–	–	–	–	–	–
20	64.6	56.7	78.5 (77.3)	0.89	5.3	13.5	88.0
40	64.4	45.5	76.4 (76.1)	<0.5	11.0	43.4	91.4
60	62.9	58.7	76.0 (75.0)	<0.5	9.4	50.1	52.0
80	48.8	59.2	78.2 (77.0)	9.6	18.0	120.8	74.8
100	–	63.4	74.5 (74.6)	24.3	30.1	243.8	100

* The first temperature was obtained during the first heating scan, whereas the temperature in parentheses corresponds to the second heating scan.

**Table 2:** Glass transition temperature and storage modulus of cured epoxy compositions containing 12.5% hydrophilic silica and different Wood’s metal mass fractions.

*w*_WM_, wt%	*G*′_25°C_, MPa	*G*_N_^0^, MPa	*T*_g,tan*δ*_, °C
0	770	33	86
20	630	20	60
40	460	18	61
60	230	3.9	58
80	830	0.4	49

**Table 3:** Average (mean) diameter for Wood’s metal particles in cured epoxy compositions containing 12.5 wt% hydrophilic silica.

*w*_WM_, wt%	*d*, µm
20	2.4 ± 0.9
40	5.3 ± 3.0
60	4.7 ± 1.9
80	1.7 ± 0.7

## Data Availability

The data presented in this study are available upon request from the corresponding author.

## References

[1] Bondareva N.S., Sheremet M.A. (2022). Heat Transfer Performance in a Concrete Block Containing a Phase Change Material for Thermal Comfort in Buildings. Energy Build..

[2] Sharshir S.W., Joseph A., Elsharkawy M., Hamada M.A., Kandeal A.W., Elkadeem M.R., Kumar Thakur A., Ma Y., Eid Moustapha M., Rashad M. (2023). Thermal Energy Storage Using Phase Change Materials in Building Applications: A Review of the Recent Development. Energy Build..

[3] Rashid F.L., Al-Obaidi M.A., Dulaimi A., Mahmood D.M.N., Sopian K. (2023). A Review of Recent Improvements, Developments, and Effects of Using Phase-Change Materials in Buildings to Store Thermal Energy. Designs.

[4] Sharma R.K., Kumar A., Rakshit D. (2024). A Phase Change Material (PCM) Based Novel Retrofitting Approach in the Air Conditioning System to Reduce Building Energy Demand. Appl. Therm. Eng..

[5] Masood U., Haggag M., Hassan A., Laghari M. (2023). A Review of Phase Change Materials as a Heat Storage Medium for Cooling Applications in the Built Environment. Buildings.

[6] Goel V., Saxena A., Kumar M., Thakur A., Sharma A., Bianco V. (2023). Potential of Phase Change Materials and Their Effective Use in Solar Thermal Applications: A Critical Review. Appl. Therm. Eng..

[7] Imran Khan M., Asfand F., Al-Ghamdi S.G. (2023). Progress in Research and Development of Phase Change Materials for Thermal Energy Storage in Concentrated Solar Power. Appl. Therm. Eng..

[8] Wang F., Pang D., Liu X., Liu M., Du W., Zhang Y., Cheng X. (2023). Progress in Application of Phase-Change Materials to Cooling Clothing. J. Energy Storage.

[9] Zare M., Mikkonen K.S. (2023). Phase Change Materials for Life Science Applications. Adv. Funct. Mater..

[10] Mehling H., Brütting M., Haussmann T. (2022). PCM Products and Their Fields of Application—An Overview of the State in 2020/2021. J. Energy Storage.

[11] Wang G., Tang Z., Gao Y., Liu P., Li Y., Li A., Chen X. (2023). Phase Change Thermal Storage Materials for Interdisciplinary Applications. Chem. Rev..

[12] Yuan K., Shi J., Aftab W., Qin M., Usman A., Zhou F., Lv Y., Gao S., Zou R. (2020). Engineering the Thermal Conductivity of Functional Phase-Change Materials for Heat Energy Conversion, Storage, and Utilization. Adv. Funct. Mater..

[13] Huang J., Luo Y., Weng M., Yu J., Sun L., Zeng H., Liu Y., Zeng W., Min Y., Guo Z. (2021). Advances and Applications of Phase Change Materials (PCMs) and PCMs-Based Technologies. ES Mater. Manuf..

[14] Yang T., King W.P., Miljkovic N. (2021). Phase Change Material-Based Thermal Energy Storage. Cell Rep. Phys. Sci..

[15] Atinafu D.G., Ok Y.S., Kua H.W., Kim S. (2020). Thermal Properties of Composite Organic Phase Change Materials (PCMs): A Critical Review on Their Engineering Chemistry. Appl. Therm. Eng..

[16] Liu Q., Zhang J., Liu J., Sun W., Xu H., Liu C. (2023). Self-Healed Inorganic Phase Change Materials for Thermal Energy Harvesting and Management. Appl. Therm. Eng..

[17] Junaid M.F., Rehman Z.U., Čekon M., Čurpek J., Farooq R., Cui H., Khan I. (2021). Inorganic Phase Change Materials in Thermal Energy Storage: A Review on Perspectives and Technological Advances in Building Applications. Energy Build..

[18] Sun M., Liu T., Sha H., Li M., Liu T., Wang X., Chen G., Wang J., Jiang D. (2023). A Review on Thermal Energy Storage with Eutectic Phase Change Materials: Fundamentals and Applications. J. Energy Storage.

[19] Glova A.D., Nazarychev V.M., Larin S.V., Lyulin A.V., Lyulin S.V., Gurtovenko A.A. (2022). Asphaltenes as Novel Thermal Conductivity Enhancers for Liquid Paraffin: Insight from in Silico Modeling. J. Mol. Liq..

[20] Bondareva N.S., Sheremet M.A. (2019). Effect of Nano-Sized Heat Transfer Enhancers on PCM-Based Heat Sink Performance at Various Heat Loads. Nanomaterials.

[21] Glova A.D., Nazarychev V.M., Larin S.V., Gurtovenko A.A., Lyulin S.V. (2023). Size Matters: Asphaltenes with Enlarged Aromatic Cores Promote Heat Transfer in Organic Phase-Change Materials. Phys. Chem. Chem. Phys..

[22] Shamseddine I., Pennec F., Biwole P., Fardoun F. (2022). Supercooling of Phase Change Materials: A Review. Renew. Sustain. Energy Rev..

[23] Zhao Y., Zhang X., Xu X., Zhang S. (2020). Research Progress in Nucleation and Supercooling Induced by Phase Change Materials. J. Energy Storage.

[24] Yu K., Liu Y., Yang Y. (2021). Review on Form-Stable Inorganic Hydrated Salt Phase Change Materials: Preparation, Characterization and Effect on the Thermophysical Properties. Appl. Energy.

[25] Zhang S., Jin Y., Yan Y. (2022). Depression of Melting Point and Latent Heat of Molten Salts as Inorganic Phase Change Material: Size Effect and Mechanism. J. Mol. Liq..

[26] Mohamed S.A., Al-Sulaiman F.A., Ibrahim N.I., Zahir M.H., Al-Ahmed A., Saidur R., Yılbaş B.S., Sahin A.Z. (2017). A Review on Current Status and Challenges of Inorganic Phase Change Materials for Thermal Energy Storage Systems. Renew. Sustain. Energy Rev..

[27] Ge H., Li H., Mei S., Liu J. (2013). Low Melting Point Liquid Metal as a New Class of Phase Change Material: An Emerging Frontier in Energy Area. Renew. Sustain. Energy Rev..

[28] Shamberger P.J., Bruno N.M. (2020). Review of Metallic Phase Change Materials for High Heat Flux Transient Thermal Management Applications. Appl. Energy.

[29] Zhu S., Nguyen M.T., Yonezawa T. (2021). Micro- and Nano-Encapsulated Metal and Alloy-Based Phase-Change Materials for Thermal Energy Storage. Nanoscale Adv..

[30] Handschuh-Wang S., Gancarz T., Uporov S., Wang T., Gao E., Stadler F.J., Zhou X. (2022). A Short History of Fusible Metals and Alloys—Towards Room Temperature Liquid Metals. Eur. J. Inorg. Chem..

[31] Huang Y., Stonehouse A., Abeykoon C. (2023). Encapsulation Methods for Phase Change Materials—A Critical Review. Int. J. Heat Mass Transf..

[32] Pasarkar N.P., Yadav M., Mahanwar P.A. (2023). A Review on the Micro-Encapsulation of Phase Change Materials: Classification, Study of Synthesis Technique and Their Applications. J. Polym. Res..

[33] Vasilyev G., Koifman N., Shuster M., Gishvoliner M., Cohen Y., Zussman E. (2020). Synergistic Effect of Two Organogelators for the Creation of Bio-Based, Shape-Stable Phase-Change Materials. Langmuir.

[34] Vasilyev G., Koifman N., Shuster M., Gishvoliner M., Cohen Y., Zussman E. (2022). Phase Change Material with Gelation Imparting Shape Stability. ACS Omega.

[35] Ilyina S.O., Gorbunova I.Y., Makarova V.V., Kerber M.L., Ilyin S.O. (2023). Self-Lubricating and Shape-Stable Phase-Change Materials Based on Epoxy Resin and Vegetable Oils. Polymers.

[36] Ilyina S.O., Gorbunova I.Y., Yadykova A.Y., Vlasova A.V., Kerber M.L., Ilyin S.O. (2024). Naphthalene-Containing Epoxy Resin: Phase Structure, Rheology, and Thermophysical Properties. Polymers.

[37] Ilyina S.O., Gorbunova I.Y., Kerber M.L., Ilyin S.O. (2025). Epoxy Resin Highly Loaded with an Ionic Liquid: Morphology, Rheology, and Thermophysical Properties. Gels.

[38] Ilyin S.O., Malkin A.Y., Kulichikhin V.G., Shaulov A.Y., Stegno E.V., Berlin A.A., Patlazhan S.A. (2014). Rheological Properties of Polyethylene/Metaboric Acid Thermoplastic Blends. Rheol. Acta.

[39] Kostyuk A.V., Ignatenko V.Y., Makarova V.V., Antonov S.V., Ilyin S.O. (2020). Polyethylene Wax as an Alternative to Mineral Fillers for Preparation of Reinforced Pressure-Sensitive Adhesives. Int. J. Adhes. Adhes..

[40] Handschuh-Wang S., Wang B., Wang T., Stadler F.J. (2023). Measurement Principles for Room Temperature Liquid and Fusible Metals’ Surface Tension. Surf. Interfaces.

[41] Gorbacheva S.N., Ilyin S.O. (2021). Morphology and Rheology of Heavy Crude Oil/Water Emulsions Stabilized by Microfibrillated Cellulose. Energy Fuels.

[42] Grace H.P. (1982). Dispersion phenomena in high viscosity immiscible fluid systems and application of static mixers as dispersion devices in such systems. Chem. Eng. Commun..

[43] Mironova M.V., Ilyin S.O. (2018). Effect of Silica and Clay Minerals on Rheology of Heavy Crude Oil Emulsions. Fuel.

[44] Yadykova A.Y., Ilyin S.O. (2022). Rheological and Adhesive Properties of Nanocomposite Bitumen Binders Based on Hydrophilic or Hydrophobic Silica and Modified with Bio-Oil. Constr. Build. Mater..

[45] Frelichowska J., Bolzinger M.-A., Chevalier Y. (2009). Pickering Emulsions with Bare Silica. Colloids Surf. A Physicochem. Eng. Asp..

[46] Björkegren S., Nordstierna L., Törncrona A., Palmqvist A. (2017). Hydrophilic and Hydrophobic Modifications of Colloidal Silica Particles for Pickering Emulsions. J. Colloid Interface Sci..

[47] Whaval S.P., Tiwari A.K. (2025). A Review on the Synergistic Effect of Various Surfactants and Silica Nanoparticles on Pickering Emulsion. J. Dispers. Sci. Technol..

[48] Malkin A.Y., Ilyin S.O., Arinina M.P., Kulichikhin V.G. (2017). The Rheological State of Suspensions in Varying the Surface Area of Nano-Silica Particles and Molecular Weight of the Poly(Ethylene Oxide) Matrix. Colloid Polym. Sci..

[49] Bao J., Zou D., Zhu S., Ma Q., Wang Y., Hu Y. (2021). A Medium-Temperature, Metal-Based, Microencapsulated Phase Change Material with a Void for Thermal Expansion. Chem. Eng. J..

[50] Lei K., Wang S., Wang Z., Wang H., Zou D. (2023). A Metal-Based Microencapsulated Phase Change Material (MEPCM) with High Thermal Reliability and Its Performance Regulation. Compos. Part A Appl. Sci. Manuf..

[51] Morgan R.J. (1979). The Effect of Thermal History and Strain Rate on the Mechanical Properties of Diethylenetriamine-cured bisphenol-A-diglycidyl Ether Epoxies. J. Appl. Polym. Sci..

[52] Ismail N.A., Shakoor R.A., Kahraman R. (2023). Corrosion Inhibition Performance of Developed Epoxy Coatings Containing Carbon Nanocapsules Loaded with Diethylenetriamine. Prog. Org. Coat..

[53] Ma C., Zhang Y., Chen X., Song X., Tang K. (2020). Experimental Study of an Enhanced Phase Change Material of Paraffin/Expanded Graphite/Nano-Metal Particles for a Personal Cooling System. Materials.

[54] Li J., Hu X., Zhang C., Luo W., Jiang X. (2021). Enhanced Thermal Performance of Phase-Change Materials Supported by Mesoporous Silica Modified with Polydopamine/Nano-Metal Particles for Thermal Energy Storage. Renew. Energy.

[55] Kalidasan B., Pandey A.K., Saidur R., Tyagi V.V. (2023). Energizing Organic Phase Change Materials Using Silver Nanoparticles for Thermal Energy Storage. J. Energy Storage.

[56] Makarova V.V., Gorbacheva S.N., Antonov S.V., Ilyin S.O. (2020). On the Possibility of a Radical Increase in Thermal Conductivity by Dispersed Particles. Russ. J. Appl. Chem..

[57] Aramesh M., Shabani B. (2022). Metal Foam-Phase Change Material Composites for Thermal Energy Storage: A Review of Performance Parameters. Renew. Sustain. Energy Rev..

[58] Li W., Wan H., Lou H., Fu Y., Qin F., He G. (2017). Enhanced Thermal Management with Microencapsulated Phase Change Material Particles Infiltrated in Cellular Metal Foam. Energy.

[59] Sabet S., Buonomo B., Sheremet M.A., Manca O. (2023). Numerical Investigation of Melting Process for Phase Change Material (PCM) Embedded in Metal Foam Structures with Kelvin Cells at Pore Scale Level. Int. J. Heat Mass Transf..

[60] Navarrete N., Mondragón R., Wen D., Navarro M.E., Ding Y., Juliá J.E. (2019). Thermal Energy Storage of Molten Salt –Based Nanofluid Containing Nano-Encapsulated Metal Alloy Phase Change Materials. Energy.

[61] Yang T., Kang J.G., Weisensee P.B., Kwon B., Braun P.V., Miljkovic N., King W.P. (2020). A Composite Phase Change Material Thermal Buffer Based on Porous Metal Foam and Low-Melting-Temperature Metal Alloy. Appl. Phys. Lett..

[62] Blanco-Rodríguez P., Rodríguez-Aseguinolaza J., Risueño E., Tello M. (2014). Thermophysical Characterization of Mg–51%Zn Eutectic Metal Alloy: A Phase Change Material for Thermal Energy Storage in Direct Steam Generation Applications. Energy.

[63] Confalonieri C., Grimaldi A.T., Gariboldi E. (2020). Ball-Milled Al–Sn Alloy as Composite Phase Change Material. Mater. Today Energy.

[64] Fukahori R., Nomura T., Zhu C., Sheng N., Okinaka N., Akiyama T. (2016). Thermal Analysis of Al–Si Alloys as High-Temperature Phase-Change Material and Their Corrosion Properties with Ceramic Materials. Appl. Energy.

[65] Kawaguchi T., Sakai H., Sheng N., Kurniawan A., Nomura T. (2020). Microencapsulation of Zn-Al Alloy as a New Phase Change Material for Middle-High-Temperature Thermal Energy Storage Applications. Appl. Energy.

[66] Liu L., Chen J., Qu Y., Xu T., Wu H., Zhou X., Zhang H. (2019). Preparation and Thermal Properties of Low Melting Point Alloy/Expanded Graphite Composite Phase Change Materials Used in Solar Water Storage System. Sol. Energy Mater. Sol. Cells.

[67] Noohi Z., Nosouhian S., Niroumand B., Timelli G. (2022). Use of Low Melting Point Metals and Alloys (Tm < 420 °C) as Phase Change Materials: A Review. Metals.

[68] Schilde C., Nolte H., Arlt C., Kwade A. (2010). Effect of Fluid–Particle-Interactions on Dispersing Nano-Particles in Epoxy Resins Using Stirred-Media-Mills and Three-Roll-Mills. Compos. Sci. Technol..

[69] Brantseva T.V., Ilyin S.O., Gorbunova I.Y., Antonov S.V., Korolev Y.M., Kerber M.L. (2016). Epoxy Reinforcement with Silicate Particles: Rheological and Adhesive Properties—Part II: Characterization of Composites with Halloysite. Int. J. Adhes. Adhes..

[70] Ying L., Wu Y., Nie C., Wu C., Wang G. (2020). Improvement of the Tribological Properties and Corrosion Resistance of Epoxy–PTFE Composite Coating by Nanoparticle Modification. Coatings.

[71] Papadopoulou A., Gillissen J.J., Wilson H.J., Tiwari M.K., Balabani S. (2020). On the Shear Thinning of Non-Brownian Suspensions: Friction or Adhesion?. J. Non-Newton. Fluid Mech..

[72] Pan D., Wang Y., Yoshino H., Zhang J., Jin Y. (2023). A Review on Shear Jamming. Phys. Rep..

[73] Vinogradov G.V. (1977). Ultimate Regimes of Deformation of Linear Flexible Chain Fluid Polymers. Polymer.

[74] Raghavan S.R., Walls H.J., Khan S.A. (2000). Rheology of Silica Dispersions in Organic Liquids: New Evidence for Solvation Forces Dictated by Hydrogen Bonding. Langmuir.

[75] Ilyin S.O., Arinina M.P., Malkin A.Y., Kulichikhin V.G. (2016). Sol–Gel Transition and Rheological Properties of Silica Nanoparticle Dispersions. Colloid J..

[76] Hyun K., Wilhelm M., Klein C.O., Cho K.S., Nam J.G., Ahn K.H., Lee S.J., Ewoldt R.H., McKinley G.H. (2011). A Review of Nonlinear Oscillatory Shear Tests: Analysis and Application of Large Amplitude Oscillatory Shear (LAOS). Prog. Polym. Sci..

[77] Crawford N.C., Williams S.K.R., Boldridge D., Liberatore M.W. (2013). Shear-Induced Structures and Thickening in Fumed Silica Slurries. Langmuir.

[78] Li B., Guo Y., Steeman P., Bulters M., Yu W. (2021). Shear-Induced Breakdown and Agglomeration in Nanoparticles Filled Polymer: The Shift of Phase Boundary and Kinetics. J. Rheol..

[79] Gorbacheva S.N., Makarova V.V., Ilyin S.O. (2021). Hydrophobic Nanosilica-Stabilized Graphite Particles for Improving Thermal Conductivity of Paraffin Wax-Based Phase-Change Materials. J. Energy Storage.

[80] Beris A.N., Tsamopoulos J.A., Armstrong R.C., Brown R.A. (1985). Creeping Motion of a Sphere through a Bingham Plastic. J. Fluid Mech..

[81] Park S.-J., Jin F.-L., Lee J.-R. (2004). Thermal and Mechanical Properties of Tetrafunctional Epoxy Resin Toughened with Epoxidized Soybean Oil. Mater. Sci. Eng. A.

[82] Hossain M.K., Himuro Y., Morita K., Nakagawa K., Matsumoto T., Fukuda K., Maschek W. (2009). Simulation of Molten Metal Penetration and Freezing Behavior in a Seven-Pin Bundle Experiment. J. Nucl. Sci. Technol..

[83] Emady H., Caggioni M., Spicer P. (2013). Colloidal Microstructure Effects on Particle Sedimentation in Yield Stress Fluids. J. Rheol..

[84] Meakin P. (1987). Fractal Aggregates. Adv. Colloid Interface Sci..

[85] Kulkarni P., Biswas P. (2004). A Brownian Dynamics Simulation to Predict Morphology of Nanoparticle Deposits in the Presence of Interparticle Interactions. Aerosol Sci. Technol..

[86] Zhang Q., Weber C., Schubert U.S., Hoogenboom R. (2017). Thermoresponsive Polymers with Lower Critical Solution Temperature: From Fundamental Aspects and Measuring Techniques to Recommended Turbidimetry Conditions. Mater. Horiz..

[87] Pasparakis G., Tsitsilianis C. (2020). LCST Polymers: Thermoresponsive Nanostructured Assemblies towards Bioapplications. Polymer.

[88] French S.J. (1936). Melting Points of Eutectics. Ind. Eng. Chem..

[89] Rehbinder P.A. (1965). Formation of Structures in Disperse Systems. Pure Appl. Chem..

[90] Uriev N.B. (2004). Physicochemical Dynamics of Disperse Systems. Russ. Chem. Rev..

[91] Gorbacheva S.N., Yarmush Y.M., Ilyin S.O. (2020). Rheology and Tribology of Ester-Based Greases with Microcrystalline Cellulose and Organomodified Montmorillonite. Tribol. Int..

[92] Ilyin S.O., Konstantinov I.I. (2022). Rheology of Highly Ordered Smectic Phases Based on Biphenyl Derivatives. J. Mol. Liq..

[93] Utracki L.A. (1991). On the Viscosity-concentration Dependence of Immiscible Polymer Blends. J. Rheol..

[94] Zhao R., Macosko C.W. (2002). Slip at Polymer–Polymer Interfaces: Rheological Measurements on Coextruded Multilayers. J. Rheol..

[95] Ilyin S.O., Makarova V.V., Polyakova M.Y., Kulichikhin V.G. (2020). Phase State and Rheology of Polyisobutylene Blends with Silicone Resin. Rheol. Acta.

[96] Ilyin S.O. (2024). Structural Rheology in the Development and Study of Complex Polymer Materials. Polymers.

[97] Morris J.F. (2020). Shear Thickening of Concentrated Suspensions: Recent Developments and Relation to Other Phenomena. Annu. Rev. Fluid Mech..

[98] Huang G.C., Lee J.K. (2010). Isothermal Cure Characterization of Fumed Silica/Epoxy Nanocomposites: The Glass Transition Temperature and Conversion. Compos. Part A Appl. Sci. Manuf..

[99] Hodge I.M. (1994). Enthalpy Relaxation and Recovery in Amorphous Materials. J. Non-Cryst. Solids.

[100] Christenson H.K. (2001). Confinement Effects on Freezing and Melting. J. Phys. Condens. Matter.

[101] Meldrum F.C., O’Shaughnessy C. (2020). Crystallization in Confinement. Adv. Mater..

[102] Ilyina S.O., Vlasova A.V., Gorbunova I.Y., Lukashov N.I., Kerber M.L., Ilyin S.O. (2023). Epoxy Phase-Change Materials Based on Paraffin Wax Stabilized by Asphaltenes. Polymers.

[103] Fenton A.M., Xie R., Aplan M.P., Lee Y., Gill M.G., Fair R., Kempe F., Sommer M., Snyder C.R., Gomez E.D. (2022). Predicting the Plateau Modulus from Molecular Parameters of Conjugated Polymers. ACS Cent. Sci..

